# The mouse gastric surface epithelial cell and its response to early *Helicobacter pylori* infection

**DOI:** 10.1080/21505594.2026.2645859

**Published:** 2026-03-13

**Authors:** Mattias Erhardsson, Licínia Santos, John Benktander, Sinan Sharba, Kaisa Thorell, Sara Lindén

**Affiliations:** a Department of Medical Biochemistry and Cell Biology, Institute of Biomedicine, Sahlgrenska Academy, University of Gothenburg, Gothenburg, Sweden; b Department of Chemistry & Molecular Biology, Faculty of Science and Technology, University of Gothenburg, Gothenburg, Sweden; c SciLifeLab, Department of Chemistry & Molecular Biology, Faculty of Science and Technology, University of Gothenburg, Gothenburg, Sweden

**Keywords:** Helicobacter pylori, gastric mucosa, surface mucus cells, foveolar cells, RNA-Seq, Laser capture microdissection, oxidative phosphorylation, interferons, glycosylation, gastric mucins

## Abstract

*Helicobacter pylori* infection is the main risk factor for gastric cancer. *H. pylori* easily develop antibiotic resistance and evade host defenses. In-depth knowledge of the first barrier that *H. pylori* encounter, the gastric surface mucus-producing epithelial cells (SMCs), may enable improved treatment and prevention. This study aimed to characterize SMC gene expression, mucus glycosylation, and identify how *H. pylori* colonization affects these parameters. The glycosylation of eight *H. pylori*-infected and eight sham control mice was characterized by mass spectrometry. SMCs from five infected and five sham control mice were extracted with laser microdissection (LCM) and sequenced with RNA sequencing (RNA-Seq). SMCs were characterized by high gene expression for proteins secreted into mucus (*Tff1*, *Gkn1*, *Gkn2*, *Psca*, and *Muc5ac)*, mitoribosome RNA, and cytoskeleton proteins. Mucin glycans were large, complex, heavily fucosylated, and dense with H-antigen motifs. Two main glycosylation pathways ending in H-antigen glycans were identified and corroborated with glycosyltransferase expression. Glycosylation was consistent between *H. pylori*-infected and sham control mice. RNA-Seq data was analysed for differential gene expression, gene set enrichment analysis, and network analysis of functionally-related genes. The analyses revealed that genes required for protein synthesis and oxidative phosphorylation were down-regulated in infected mice. Most up-regulated genes were either interferon-stimulated genes or able to induce interferon production themselves. Depletion of Nkx6-3 occurred in the infected mice, indicating initiation of a pre-cancerous cascade. LCM RNA-Seq of SMCs was thus feasible and enabled characterization of the SMC and definition of a gene set showing how *H. pylori* infection affects SMCs.

## Introduction

*Helicobacter pylori* is a helical-shaped, gram-negative, carcinogenic bacterium that colonizes half of all human stomachs [1]. *H. pylori* infection is the primary risk factor for gastric cancer [2], which is the fourth leading cause of cancer mortality world-wide [3]. Chronic gastritis and gastro-duodenal ulcers can also be caused by *H. pylori* infection [2].

A wide assortment of virulence factors and strategies allow *H. pylori* to circumvent the host immune response in order to establish life-long colonization [4]. One of the challenges in treating *H. pylori* infection is its plastic genome and high propensity to develop antibiotic resistance [5]. The widespread use of antibiotics has thus contributed to the emergence of antibiotic-resistant strains of *H. pylori*, making widespread eradication of *H. pylori* through antibiotics alone unfeasible [6]. This highlights the need for alternative treatment strategies and a better understanding of the mechanisms underlying *H. pylori* pathogenesis and colonization.

The mucus layer is the first layer of defense and protects the host through several mechanisms, among them by acting as a physical barrier between the bacteria and the gastric epithelium. The mucus layer consists mainly of water and mucins, and mucin glycosylation play crucial roles in defending against *H. pylori* infection [7–9]. A healthy stomach expresses 3 different mucin genes: MUC1, MUC5AC, and MUC6. MUC1 is a transmembrane mucin which can be shed when *H. pylori* bind to the glycans [9]. The secreted mucus layer is segmented so that MUC6 fills the glands, while MUC5AC makes up surface mucus, as well as a mixture of the two where mucins meet in the glandular isthmus [10]. MUC5AC is expressed by the gastric surface mucus-producing epithelial cells (SMCs), and the protective mechanisms provided by the mucus produced by these cells must be overcome for *H. pylori* to be able to infect the host. These SMCs can also be called foveolar cells, but this term could also include the deeper, glandular MUC6-expressing cells. Another alias for the MUC5AC-expressing cell type is pit cell [11], as these cells are found on both the surface and in the pit, i.e. the upper region of the gastric glands/gastric units. SMCs was the nomenclature of choice for this study.

Mice infected with *H. pylori* strain SS1 is a widely-used animal model to study *H. pylori* pathogenesis, treatment [12], and immunization [13]. Other experiments with SS1-infected mice include the discovery of an immune regulatory mechanism where blocking programmed cell death ligand 1 (PD-L1), thus preventing stimulation of programmed death 1 receptors (PD-1) by gastric dendritic cells, worsened *H. pylori*-induced gastritis [14]. *H. pylor*i induced inhibition of mucin production in SMCs, which likely contributes to long-term *H. pylori* colonization, has also been shown using the SS1-infection model [15].

Single-cell RNA sequencing (scRNA-Seq) has been instrumental in characterizing cell-type-specific responses in the gastric mucosa. In humans, scRNA-Seq of immune cells revealed expansion of NKp44^+^ ILC3s, CD11c^+^ myeloid cells, and activated CD4^+^ T and B cells in *H. pylori*–colonized compared to non-colonized individuals. In mice, analysis of gastric epithelial cells identified spasmolytic polypeptide – expressing metaplasia (SPEM) cells lacking *Gif* gene expression, and further demonstrated that *H. pylori* preferentially adheres to SMCs, likely due to chemotaxis toward urea produced by these cells [16–18].

Laser capture microdissection (LCM) offers another precise approach to studying specific gastric cell populations. LCM followed by microarray analysis revealed over 1000 differentially-expressed genes in parietal, chief, and mucus-producing cells from *H. pylori*–infected mice, including up-regulation of prostaglandin E₂ receptor 4 (EP4). Similar approaches identified *Calgranulin A* up-regulation as a marker of MALT lymphoma in gastric lymphoid aggregates and provided evidence that SPEM represents an early stage in gastric tumorigenesis [11,19–21]. Compared to microarrays, RNA sequencing (RNA-Seq) offers transcriptome-wide coverage, both relative and absolute expression levels instead of only relative, and a broader dynamic range [22]. However, the RNA-Seq-based transcriptional profile of normal SMCs during *H. pylori* remains unexplored. The present study addresses this gap, providing insight into early epithelial responses to *H. pylori* infection.

While gastric mucin glycosylation has been studied in mice [23–25] and humans [26], recent advances in glycobioinformatics enables more statistically sound and biologically relevant analysis of glycan data than previously possible, including motif-level differential glycomics [27].

The overall aims of this study were to characterize the normal SMC gene expression and mucus glycosylation as well as determine how *H. pylori* colonization affects these parameters, thereby advancing our understanding of *H. pylori* colonization and pathogenesis. In doing so, we also developed LCM-RNA-seq as a methodology for studying host responses to infection in-vivo, identified key mucin glycan biosynthesis pathways by integrating glycomics with glycosyltransferase transcriptomics and validated some differentially-expressed genes using immunofluorescence.

## Materials and methods

### Overview

Reporting of this study followed the ARRIVE 2.0 reporting guidelines [28]. The reporting checklist is provided in supplemental file 10 which can be accessed at the following link: https://github.com/mattias-erhardsson/lmpc-infection-rnaseq/tree/main/Virulence%20supplemental%20files.

**Figure 1.**
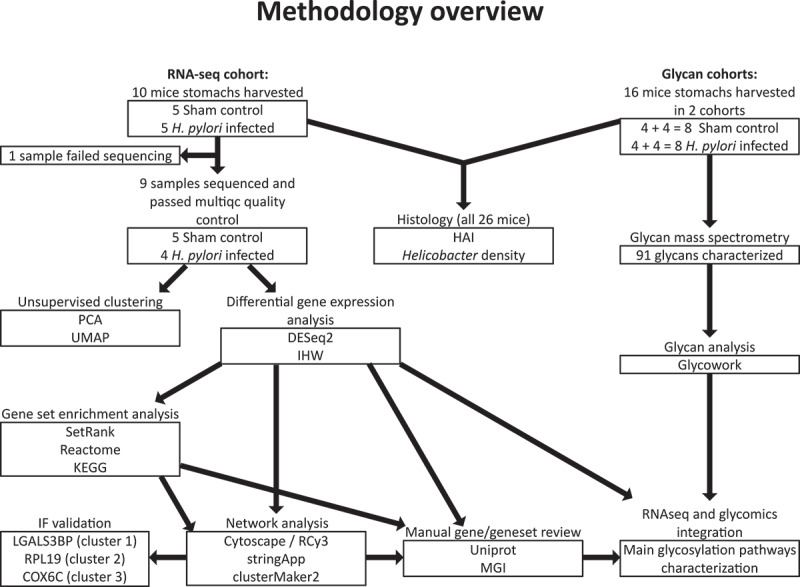
Flowchart of the path from harvested mouse stomachs to analysed gene expression data. PCA = Principal Component Analysis. UMAP = Uniform Manifold Approximation and Projection. DESeq2 = the R package DESeq2. IHW = independent Hypothesis Weighting. HAI = Histological Activity Index. SetRank = the R package SetRank. Reactome = the reactome pathway database was used in the SetRank analysis. KEGG = the Kyoto Encyclopedia of Genes and Genomes database was used in the SetRank analysis. Cytoscape/RCy3 = the R package RCy3 was used for programmatic network analysis in the software Cytoscape. stringApp = the app stringApp was used to annotate genes in the Cytoscape analysis with interactions from the string database. clusterMaker2 = the app clusterMaker2 was used to cluster gene interactions in the Cytoscape analysis. UniProt = the UniProt database accessed with the web interface. MGI = the Mouse Genome Informatics database accessed with the web interface. Glycowork = the Glycowork python package used for glycomics analyses. IF = Immunofluorescence.

See Figure 1 for an overview of the methodology employed in this article. Ten mouse stomachs were harvested for the RNA-Seq cohort and 16 for the glycan cohort.

In the RNA-Seq cohort, corpus SMCs were collected using LCM, the RNA was extracted from the cells, and the isolated RNA sequenced. Nine of the samples were successfully sequenced and passed the MultiQC quality control. They were analysed with multiple bioinformatics tools for differential gene expression, gene set enrichment, and network analysis. A manual review of bioinformatics databases was also conducted for salient genes and gene sets. Histological differences between infected and sham control mice were clear in pilot experiments in our laboratory conducted at the same time points and with the same group sizes as the RNA-Seq cohort, which formed the basis for group sizes. Furthermore, group sizes of five are sufficient for RNA-Seq even if one to two samples would fail sequencing [29].

For both the RNA-Seq and glycan cohorts, all stomachs were assessed histologically for gastritis (histological index) and *H. pylori* density using fluorescent in situ hybridization (FISH).

### Ethical approval

In Sweden, animal experiments require approval from regional ethics committees coordinated by the Swedish Board of Agriculture. Animal suffering is weighed against scientific value and the possibility of using non-animal alternatives. Ethical approval was granted by **Göteborgs Djurförsöksetiska Nämnd** (Ethic No. 52–2021).

### Animals

#### RNA-Seq cohort

Ten male C57BL/6Ntac mice (Taconic Biosciences, Denmark), 6-weeks old and specific-pathogen-free, were kept at the Laboratory for Experimental Biomedicine, University of Gothenburg. The animals were kept in individually ventilated cages with 12-hour light/dark cycles and had free access to food, water, and nesting material. All five mice within each group were housed in the same cage to enable social interaction while preventing cross-contamination of *H. pylori*. The cages were kept in the same room. No inclusion or exclusion criteria were set, apart from the ethical approval-related endpoints showing general symptoms (matte fur, sitting still, or weight loss of 10% or more – none of these occurred during the experiment). When sacrificed, the mice were sedated with isoflurane followed by cervical dislocation. The same mice were also used in another 2025 study [30].

#### Glycan cohorts

A further two cohorts with a total of 16 C57BL/6Ntac mice were kept and sacrificed according to the description above. There were eight mice in each cohort, of which four were *H. pylori* infected and four were sham control. The same mice were also used in another 2025 study [30].

### Experimental H. pylori infection

A stock culture of *H. pylori* strain SS1 was kept at −80°C in Brain-Heart infusion (BHI) broth (CM1135, Oxoid) containing 20% glycerol. Bacteria from the stock culture was brought up and cultured on Brucella agar (CM0169, Oxoid) supplemented with 10% horse blood in microaerobic conditions for four days. The bacteria were then transferred and cultured overnight in BHI with 10% fetal bovine serum (FBS) in microaerobic conditions. Bacteria were then suspended in phosphate-buffered solution (PBS) to a 600 nm optical density (OD_600_) of 1.0. Quality control involved inspecting the suspension under a microscope to ensure the viability of bacteria and re-culturing the suspension on agar for four days and counting colony forming units (CFUs). The mice were infected through oral gavage with the *H. pylori* SS1 suspension, two doses of 200 µL (3.5 ×10^7^ CFU/mouse) given one day apart when mice were eight weeks old. The sham controls were given a corresponding sham treatment of oral gavages with 200 µL PBS. The mice were harvested two weeks after the first *H. pylori* infection dose.

### Snap-freezing tissue embedded in optimal cutting temperature compound (OCT)

**Figure 2.**
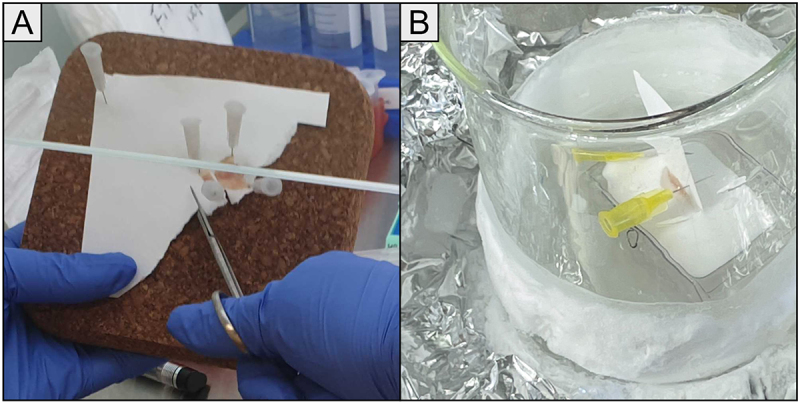
Orienting and embedding gastric tissue in OCT. (A): The stomach was opened at the curvature major and laid out on filter paper with the lumen facing up. Needles in the sides of the forestomach and antrum were used to keep the tissue flat on the tissue paper. A longitudinal strip for OCT embedding slightly to the side of the middle (curvature minor) was then cut from the antrum to the forestomach.(B): Needles in the filter paper were used to balance the tissue on its side, with the previously cut side facing down in the mold. OCT was carefully poured to avoid bubbles. The mold was transferred with forceps to a prepared beaker enclosed with aluminum foil containing a mixture of dry ice and Cryospray, which rapidly froze the OCT.

Harvested stomachs were pinned to filter paper with the lumen facing up, using needles piercing the edges of the tissue at the forestomach and antrum to keep the tissue flat and spread out against the filter paper. A longitudinal strip, encompassing forestomach to antrum, was cut out slightly to the side of the middle/curvatura minor of the harvested stomach (Figure 2(A)).

Correct orientation of the tissue when embedded into OCT was crucial for the downstream LCM. To achieve proper orientation, the cut edge was placed facing down in the embedding mold by balancing the tissue stuck to filter paper on its side, using two needles inserted into the tissue paper above to balance the tissue on the cut side (Figure 2(B)). OCT was carefully added to the mold with balanced tissue to avoid air bubbles. The filled mold was then snap-frozen by transferring the mold to a beaker with a mixture of dry ice and Cryospray Aerosol (Article number 00211, HistoLab, Gothenburg, Sweden). To minimize RNA degradation, the protocol was optimized so that it took at most ten minutes between the mouse being sacrificed to the gastric strip being frozen in OCT. Frozen molds were sealed together with desiccant bags in 50 mL centrifuge tubes and immediately put on dry ice until they could be stored at −80°C.

### Formalin-fixed paraffin-embedded (FFPE) histological assays

Gastric corpus tissue for FFPE histological assays were collected in parallel to the OCT-embedded tissue from the same stomachs. The tissue was fixed in buffered formaldehyde 4% aqueous solution (pH 7.0 ± 0.2, 9713.100, VWR) at harvest and embedded in paraffin.

Gastritis was assessed on Hematoxylin and Eosin (H&E) stained slides. Sectioned tissue (4 µm) was deparaffinized, hydrated, and stained first with hematoxylin, and then after a wash in water stained with eosin. The slides were washed again and then dehydrated with a series of baths with increasing concentrations of ethanol, then isopropanol, and finally xylene. Mounted slides were scored blinded according to the Histological Activity Index (HAI) [31].

Immunofluorescence (IF) was used to validate RNA-Seq results on a protein level. Slides were sectioned (4 µm), deparaffinized, and hydrated. Antigen retrieval was performed by boiling slides in 10 mM citric acid buffer (pH 6) with 0.05% Tween 20 (v/v) for 20 minutes. Slides were allowed to cool for 30 minutes before drawing hydrophobic barriers around the tissues. Tissues were washed thrice in wash buffer (0.5% BSA (m/V) + 0.05% Tween-20 (V/V) in PBS) with a 1-minute incubation per wash, permeabilized (10-minute incubation with 0.5% BSA (m/V) + 0.5% Tween-20 (V/V) in PBS), washed as before, incubated with blocking buffer (1% BSA (m/V) + 5% Normal Goat Serum (V/V) + 0.01% Tween-20 (V/V) in PBS) for 1 h, incubated with primary antibody overnight, washed three times with wash buffer with 5 minute incubations, then incubated with secondary antibody for 1 h, washed again with three 5-minute wash buffer washes, and then an extra triple wash with PBS. 200 µL liquid was added to the tissues in all steps after drawing hydrophobic markers (washes, permeabilization, blocking, primary antibody incubation, secondary antibody incubation), and the liquids were removed by tilting the slides in an incubation chamber. All incubations were performed at room temperature (except primary antibody incubation which was at 4°C) and in the dark. The primary antibodies were RPL19 (Rabbit Polyclonal Antibody diluted 1:1000, article number PA5-101384, Fisher Scientific, Waltham, USA), LGALS3BP (Recombinant Rabbit Monoclonal Antibody diluted 1:100, article number MA5-50412, Fisher Scientific, Waltham, USA), and COX6C (Recombinant Rabbit Monoclonal Antibody diluted 1:50, article number MA5-63536, Fisher Scientific, Waltham, USA). The secondary antibody for all three primary antibodies was a Goat anti-Rabbit Alexa Fluor™ 594 diluted 1:200 (article number A-11037, Fisher Scientific, Waltham, USA).

Slides were blinded, and for each slide the SMCs in the gland with the most intense signal was measured with Fiji [32] to a depth of approximately 70 μm from the surface and the mean signal intensity of this gland used as the readout. For the antibodies against cytoplasmic proteins (LGALS3BP and RPL19) the nuclei were excluded from the region of interest (ROI), while the ROI included areas with DAPI signal for the mitochondrial antibody (COX6C) since most of the signal was expected to be in close proximity to the nuclei and thus coincide with much of the DAPI signal.

Visualization of bacteria was conducted on FISH slides with two probes. The probes were EUB 338 labeled with Cy3.5 (5’GCTGCCTCCCGTAGGAGT3’) to detect all bacteria, while the other probe Alexa 488 (5’ TCTCAGGCCGGATACCCGTCATAGCCT3’) was specific for *Helicobacter* spp. Slides were deparaffinized, hydrated, washed with PBS, and air-dried. Dried slides were hybridized with a solution containing 40% (v/v) formamide, 0.1% sodium dodecyl sulfate, 0.9 M NaCl, 20 mM Tris-HCl (pH 7.4) and 10 ng/µL probe. Slides were incubated for 18 h at 37 °C in a dark humidified chamber with 40% (v/v) formamide. After incubation, the slides were washed in a solution containing 0.9 M NaCl and 20 mM Tris-HCl (pH 7.4) for 20 minutes at 50°C. Slides were then dipped in ultrapure water and air-dried in darkness. Dried slides were mounted with Prolong Gold anti-fade reagent and DAPI (Thermo Fischer Scientific). Blinded counting of *Helicobacter* bacteria was conducted in the pits since this reduces the risk of falsely low counts due to the potential loss of surface mucus during processing. Counting was done with an Eclipse 90i fluorescence microscope (Nikon, Tokyo, Japan). The results show the average counts in five 40x-magnified fields.

Differences in histological assays between the two groups were tested with Mann-Whitney U, correlations between IF signal intensity and RNA-Seq gene expression levels were tested with Spearman Rank Correlation, and the *p*-values adjusted with the Benjamini-Hochberg method [33] in the R programming language [34].

### Slide preparation for LCM

RNA degradation is slowed in fresh-frozen tissue stored at −80°C, yet not stopped. The median number of days it took between harvest and sectioning for the OCT-embedded tissue analysed in this article was eight, and within a range of six to nine days. All solutions and materials that encountered the tissue from this step and onwards were prepared to be RNase-free.

OCT-embedded tissue was sectioned to ten micrometer sections in a −20°C Cryostat and transferred to PEN-membrane slides (article number 415,190-9081–000, Carl Zeiss, Oberkochen, Germany). A pilot LCM with subsequent RNA-extraction of 12 sections yielded 15 ng purified RNA available for sequencing after quality control. Based on this pilot, we sectioned three slides with at least four sections on each slide for every sample. Slides were air-dried for two minutes in the cryostat after sectioning and then transferred to centrifuge tubes with desiccant bags, in which they were briefly stored at −80°C before staining.

Slides were stained with 1% Cresyl Violet (article number 10,045,203, Fisher Scientific, Waltham, USA) using ice-cold solutions with the following steps performed inside a hood. Slides were first transferred from the centrifuge tubes to a 70% ethanol bath for fixation lasting two minutes. The slides were then put on a staining rack and stained with 1% Cresyl Violet with 200 μL of the solution applied directly to each sample using a pipette. The staining solution was rinsed away with 70% ethanol after staining for 30 seconds. After rinsing, the slides were dipped in a series of baths consisting of 70% ethanol, 100% ethanol, and then another bath of 100%. The slides were then air-dried for one minute on a paper towel, taking care not to let the paper touch the tissue or the membrane. Ensuring a quick staining in cold liquids, followed by maximally dehydrating the samples was likely crucial to avoid obliteration of RNA. Finally, the slides were put in new centrifuge tubes with desiccant bags, and placed in dry ice before swiftly being stored at −80°C.

### LCM

SMCs were collected using LCM with a PALM MicroBeam (Carl Zeiss, Oberkochen, Germany) at the Center for Cellular Imaging, Sahlgrenska Academy, Gothenburg, Sweden.

**Figure 3.**
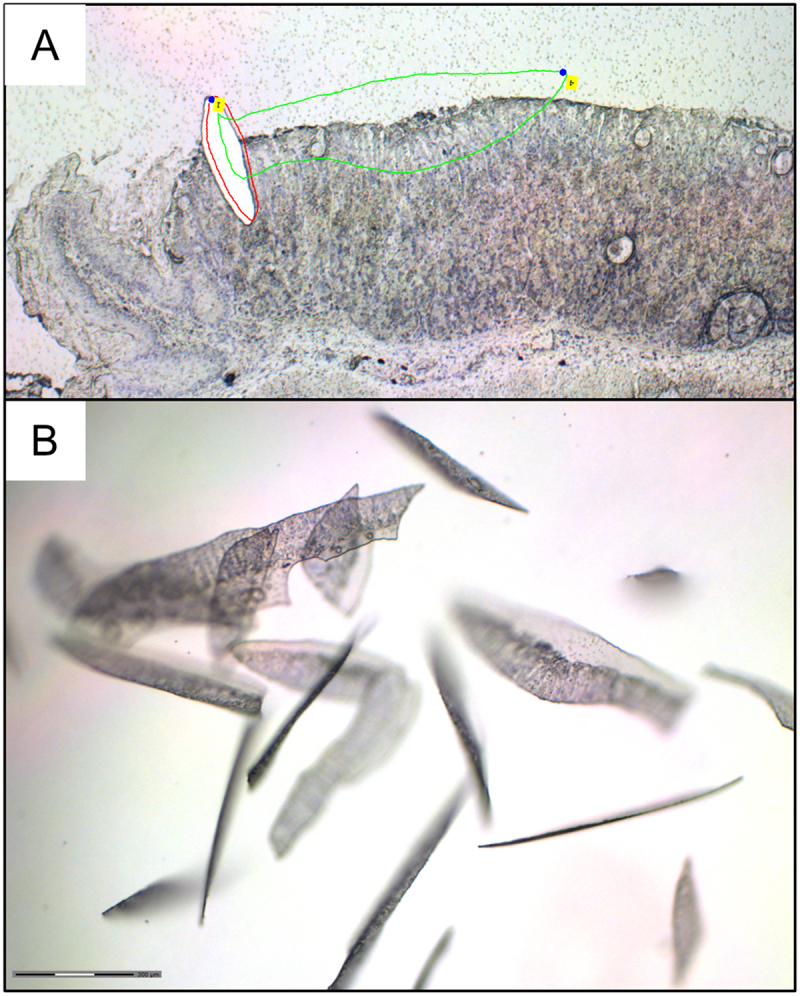
LCM collection of SMCs. A: Micrograph example of LCM cutting area. Areas for cutting were drawn starting from the forestomach transitional area as a navigational landmark. The transitional area itself (red outline) was removed but not collected. The green outline was one of the drawn areas of SMCs, which would later be cut and catapulted. The deeper layer containing parietal cells was avoided. Most cut areas contained cell-free parts of the membrane to minimize RNA damage from the laser. B: Micrograph of a collection cap after several areas had been catapulted.

The slides were thawed to room temperature while sealed inside the centrifuge tubes. To ensure that we collected SMCs from the corpus we used the forestomach transitional zone as a reference point and stained the tissue with cresyl violet to avoid parietal cells (Figure 3(A)). The collection of SMCs was carried out across the entire corpus, with the exception of the first few crypts in the forestomach-corpus transitional zone as this area differs from the rest of the corpus [31]. We also avoided the corpus-antrum transitional zone to minimize the risk of collecting antral cells. LCM for each sample took four hours, but the staining process dehydrates the tissue so RNA damage caused by the extended exposure to room temperature was minimal. Cells were cut with the 20x objective using the settings cutting speed 70, cutting energy 44, focus 74, and cutting laser function JointCut. Cut areas were catapulted with the 5x objective and catapult energy 100. Catapulting was performed in cell-free areas wherever possible, and to achieve this, approximately one-third of the collected area consisted of cell-free PEN membrane on the luminal side of the epithelium. Catapulted areas were collected in AdhesiveCap 500 clear (article number 415,190-9211–000, Carl Zeiss, Oberkochen, Germany) (Figure 3(B)). Samples in adhesive caps were then lysed by adding 350 μL of Buffer RLT from RNeasy Micro Kit (article number 74,004, Qiagen, Hilden, Germany) containing β-Mercaptoethanol to the tubes, vortexing and then incubated upside-down for 30 min. Lysates were then spun down for 10 minutes at 7300 rcf and stored at −80°C.

### RNA extraction

RNA was extracted using RNeasy Micro Kit (article number 74,004, Qiagen, Hilden, Germany). After thawing the lysed samples, the QIAGEN protocol “Total RNA Isolation from Microdissected Cryosections” was followed, starting from step five (RNeasy® Micro Handbook 04/2003, pp 20). Two μL of purified RNA were aliquoted for quality control. RNA samples were put on dry ice immediately after aliquoting and then stored at −80°C.

### RNA quality control and sequencing

Initial quality control of purified RNA Integrity Number (RIN-values) was performed with TapeStation on High Sensitivity RNA ScreenTape (Agilent, Santa Clara, USA) according to the manufacturer’s instructions.

Samples were shipped to National Genomics Infrastructure (NGI), Solna, Sweden for further quality control and subsequent sequencing. The second round of quality control was performed using Bioanalyzer (Agilent, Santa Clara, USA).

Libraries were prepared with SMARTer Total Stranded RNA-Seq, Pico input mammalian – V3 (Takara Bio, Kusatsu, Japan). Validation of constructed libraries were performed with Fragment Analyzer (Agilent, Santa Clara, USA).

Samples with successfully constructed libraries were sequenced with NovaSeq 6000 (Illumina, San Diego, USA). We aimed for a minimum sequencing depth of 30 million reads for each sample.

### Bioinformatics pipeline of fastq-files to gene counts

The nf-core/rnaseq [35] analysis pipeline was run by NGI at Uppsala Multidisciplinary Center for Advanced Computational Science, Uppsala, Sweden (UPPMAX).

### Analysis of gene expression data

Gene expression analysis consisted of three main approaches:

1. Network analysis of gene interactions between significantly differentially-expressed genes.
2. Gene set enrichment analysis of all genes ranked by adjusted *p*-values.
3. Manual review of significantly differentially-expressed genes with the most extreme fold changes and/or expression levels.

The programmatic parts of the analysis was performed using R 4.3.2 [34]. Figure 1 summarizes the analysis process.

First, counts from the bioinformatics pipeline were imported with tximport [36].

Differential gene expression was determined with DeSeq2 and *p*-values adjusted with independent hypothesis weighting (IHW) [37,38]. Clustering of gene expression was investigated with Principal Component Analysis (PCA) [39] and Uniform Manifold Approximation and Projection (UMAP) [40].

Genes were annotated with gene sets from Reactome and Kyoto Encyclopedia of genes and Genomes (KEGG) [41,42]. To achieve as complete annotation as possible to Reactome, the union of annotation with both biomaRt [43,44] and AnnotationDbi [45] was used to annotate the genes with Reactome gene sets. With biomaRt, the Ensembl version 109 and Reactome release 85 were used. With AnnotationDbi, reactome.db version 1.84.0 was used, which corresponds to Reactome release 84. KEGGREST was used to annotate the genes with KEGG release 107. Gene set enrichment analysis was performed with SetRank [46]. SetRank was run in ranked mode, with the gene list ranked by adjusted *p*-value from Deseq2/IHW. SetRank accounts for the overlap between gene sets, which was also used to adjust the *p*-values together with a multiple testing adjustment using the Holm method [46]. The background gene list consisted of all genes detected among the samples (genes with a count > 0).

Associations between significant genes and gene sets were explored programmatically in CytoScape using R package RCy3 with STRING and ClusterMaker2 apps installed [47–50]. STRING database version 12 was used for annotation. Data wrangling and visualization was facilitated with tidyverse packages [51].

Manual review of interesting genes and gene sets was performed in Uniprot and MGI, as well as by following the links provided in these database entries [52,53].

Marker genes were gathered from both PanglaoDB [54] and a scRNA-Seq dataset from mouse stomachs [17]. Marker genes from PanglaoDB were filtered based on the supplied variables canonical marker = 1, and specificity_mouse = 0, to maximize specificity. This gene list was supplemented with the marker genes found in the supplemental data of the aforementioned scRNA-Seq. All marker genes were selected for cell types which could be expected to be present in the collected tissue. Finally, marker duplicate genes which were assigned to multiple cell types in this selection were removed to further increase specificity. Before this removal of duplicates, the metaplastic cell marker genes were manually adjusted by removing *Muc6* [17], since this would otherwise overlap with the only marker gene for the important cell type mucus neck cells. The data-driven approach with strict filtering to maximize specificity, resulted in exclusion of some canonical marker genes that lacked sufficient specificity, e.g. *Cd11c* (also known as *Itgax*) a dendritic cell marker, which was also annotated as a canonical marker for macrophages [54].

### Glycan analysis

Crude mucin extractions on corpus tissue (i.e. not LCM SMCs), followed by dot blot, β-elimination and LC-MS, were conducted as described previously [55].

Glycomics analyses were performed with glycowork [56] in Jupyter [57] running Python 3 [58], as well as hierarchical agglomerative clustering (HAC) in R [59] based on glycan relative abundances. The HAC was performed with default settings (Euclidean distance metric and complete linkage) with the package pheatmap [59].

Glycans were visualized according to the Symbol Nomenclature for Glycans (SNFG) guidelines [60].

### Randomization and blinding

Harvesting and LCM was carried out sequentially, with the sham control mice first and the infected mice last. All other steps were pseudorandomized through no order being applied, and consequently were carried out in different orders.

Histological assessments were blinded during scoring and signal quantification.

## Results

### H. pylori was only detected in infected mice, and infected samples had more inflammation than sham control mice

**Figure 4.**
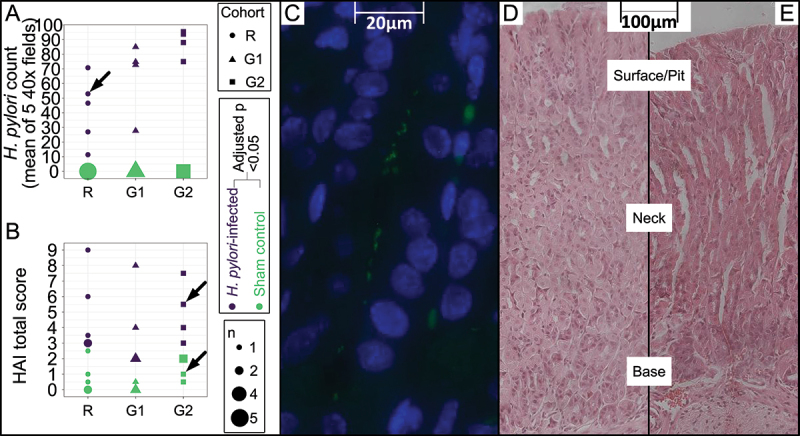
Histological assessments of H. pylori density and inflammation in H. pylori- and sham control mice.mice.The RNA-Seq cohort was denoted as r, while the two glycan cohorts were denoted G1 and G2. A: Dotplot of H. pylori counts in gastric corpus. Slides were stained with FISH-probes for bacteria and H. pylori spp. The mean Helicobacter counts of 5 fields at 40x magnification was used. Counting was performed blinded. *n* = Number of datapoints on each coordinate of the graph to account for overlapping datapoints. The arrow points to the count obtained for the photomicrograph shown in panel C. B: Dotplot of histological activity index (HAI) total scores. Scoring was performed blinded on H&E-stained slides. Infected mice had significantly (adjusted *p*-value < 0.05, Mann-Whitney U) higher HAI total scores than sham control mice. *n* = Number of datapoints on each coordinate of the graph to account for overlapping datapoints. The arrows point to the scores obtained for the photomicrographs shown in panel D and E. C: Gastric section of H. pylori-infected mouse at 14 days after infection. H. pylori was visualized with a Helicobacter fluorescent probe (green) and nuclei of cells with DAPI (blue) at 40x magnification. D and E: Gastric section stained with H&E from a sham control (d) and an H. pylori-infected mouse (e), 20x magnification. The three major layers of epithelial cells surface/pit, neck, and base are marked.

Infection status and histological activity index (HAI) for the mice were previously reported as part of a larger cohort [30]. In Figure 4, we summarize the data for animals included in this study. Infection status was confirmed by FISH using a *Helicobacter*-specific probe (Figure 4(A) and (C)). *H. pylori*-infected mice obtained higher histological activity index (HAI) scores than their sham control counterparts (Figure 4(B, D and E)). Inflammation, characterized by the presence of leukocytes in the submucosa, was the parameter that contributed the most to the scores in infected mice. Histological changes were moderate (HAI ranged 2–9 in infected mice and 0–3 in sham control mice, theoretically possible maximum score in HAI was 24), and the surface epithelium was relatively intact with no sign of infiltration of other cell types.

### RNA yield and quality from cells collected with LCM were sufficient for RNA-Seq, without amplification prior to library prep

The amount of purified RNA obtained from the 12 tissue sections processed from each mouse ranged from 16.71 to 128.58 ng from a collected area of 1.68 - 6.05 mm^2^. There was a strong correlation between collected area and quantity of purified RNA (Supplemental Figure S1). Both the quality and quantity of RNA were sufficient for the kit *SMARTer Total Stranded RNA-seq, Pico input mammalian – V3* (Supplemental Figure S1) which recommend a minimum DV200 > 50% and input amount > 10 ng.

RNA-Seq on purified RNA was successful for all samples except one from the infected group. This sample did not yield a successful library and thus could not be sequenced. We assessed that all sequenced samples passed quality control with MultiQC [61]. The mean sequencing depth was 60.9 million reads (standard deviation 6.87), and all sequenced samples were above our desired minimum sequencing depth of 30 million reads.

### LCM captured SMCs with high precision

The histology-based cellular collection method ensured that a clearly visible transition to deeper tissue where non-SMC cell types are present could be avoided. Our hypothesis was that this should entail a high purity of SMCs in sequenced material. Therefore, we investigated the gene expression of marker genes for cell types which could either be present due to cutting too deep into the mucosa, or the presence of non-SMC cell types in this outer mucosal layer. See Supplemental Table S2 and Supplemental Table S3 for details of the resulting marker genes.

**Figure 5.**
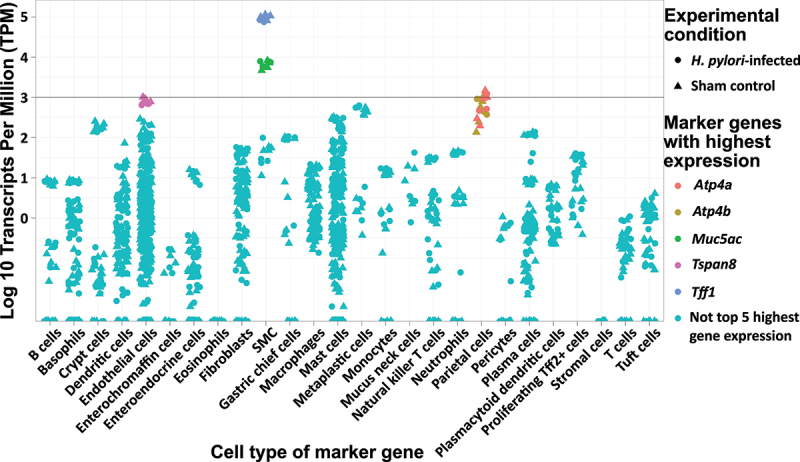
Horizontally jittered dotplot of absolute marker gene expression. Marker genes for cell types that could be present in collected tissue were chosen. The absolute gene expression was quantified as transcripts per million (TPM) and plotted on a 10-logarithmic scale since low expression genes would otherwise be drowned out by the most highly-expressed genes. The top 5 genes in terms of gene expression were highlighted through color annotation, where the gastric surface mucus-producing epithelial cell (SMC) marker gene Tff1 dwarfed the gene expression of all other marker genes. These 5 genes were also the only genes with a TPM > 1000 in at least 1 mouse (horizontal line).

Visualization of the Transcripts per million (TPM) for the marker genes showed high expression of SMC marker genes *Tff1* [17,54] and *Muc5ac* [17,54], low expression of parietal cell marker genes *Atp4a* [17] and *Atp4b* [17,54] (sub-units of gastric hydrogen potassium ATPase) as well as endothelial marker gene *Tspan8* [54], and a negligible expression of other marker genes (Figure 5).

While the gene expression levels of other marker genes were small, there was a significant difference in gene expression (adjusted *p*-value < 0.05) between infected and sham control mice for four of the marker genes, namely *Zbp1* [54] (plasma cells), *Tet2* [54] (monocytes), *Xdh* [54] and *S100a13* [54] (both for endothelial cells). There were no differences between infected and sham control groups for the expression levels of the other 11 cell markers for plasma cells, the two other cell markers for monocytes, or the 42 other cell markers for endothelial cells. The search function in PanglaoDB web interface showed that the plasma cell marker *Zbp1* is also present in cell clusters annotated as epithelial cells [54]. Furthermore, the PanglaoDB web interface showed that monocyte marker *Tet2* is present in several different cell clusters of mucosal epithelia, and endothelial markers *Xdh* and *S100a13* are present in many cell types which include epithelial and mucus producing cells [54]. Finally, the log2 fold changes for the endothelial cell markers *Xdh* and *S100a13* were opposite of each other, where *Xdh* was up-regulated while *S100a13* was down-regulated with infection. Thus, we conclude that a vast majority of the cells captured with LCM are SMCs.

### SMC gene expression was dominated by genes coding for secreted mucus proteins, mitochondrial function, and cellular structure, and mucin glycosylation was characterized by large and complex fucosylated O-glycans

We next investigated what signifies the typical gene expression of SMCs. The genes were ordered by the mean TPM in sham control mice. Genes were filtered based on the lowest expression of *Muc5ac* among sham control mice as a lower limit (Table 1). *Tff1* was the most highly-expressed gene in normal SMCs, and *Tff1* was also one of the genes coding for proteins secreted into the mucus. Products of the highly-expressed *Gkn2*, *Gkn1*, *Psca*, *Muc5ac, and Lgals2* have also been shown to be secreted into mucus [64,72,73]. The genes *mt-Rnr1*, *mt-Rnr2*, *mt-Atp6*, and *mt-Co1* were also highly expressed, and the products of these genes are known to be integral for mitochondrial functions including oxidative phosphorylation (OXPHOS) [52]. Finally, *Krt8*, *Krt19*, and *Actg1* were highly-expressed genes with known structural functions [52].

**Table 1.** Genes with the highest gene expression in the normal sham control mouse gastric corpus SMCs. The genes were ordered by the mean TPM in sham control mice. The filtering approach was to take all genes with a mean TPM at least as high as the lowest TPM of Muc5ac across all samples (TPM ≥ 4690). 18 genes remained after filtering. Genes marked with # (Tff1 and Muc5ac) were annotated as both canonical marker genes in PanglaoDB, as well as marker genes in Bockerstett et al. 2020 [17]. Genes marked with * (Gkn2, Gkn1, Psca, and Lgals2) were assigned as marker genes in Bockerstett et al. 2020 [17], but were annotated as non-canonical marker genes in PanglaoDB.

Gene	Mean TPM in sham control mice	Secreted to mucus	Mitochondrial function	Structural function
*Tff1*^*#*^	99264	Secreted [62]		
*mt-Rnr1*	96937		Mitoribosome 12S rRNA [63]	
*mt-Rnr2*	61903		Mitoribosome 16S rRNA [63]	
*Yam1*	56432			
Gkn2*	38260	Secreted [64]		
Gkn1*	37150	Secreted [64]		
*Gm26917*	22724			
Psca*	17950	Secreted [65]		
*mt-Atp6*	13874		OXPHOS complex V subunit [66]	
*Dpcr1*	10123	Also known as Mucl3, but not known to be secreted [52]		
*Krt8*	6670			Type II cytoskeleton keratin [67]
*Muc5ac*^*#*^	6613	Secreted [10]		
*mt-Co1*	6243		OXPHOS complex IV subunit [68]	
*Mal*	5348			Potential organizer of membrane lipid rafts [69]
Lgals2*	4948	Secreted [70]		
*Actg1*	4887			Cytoskeleton γ-actin, which in epithelial cells is localized in the apical side [71]
*Krt19*	4833			Type I cytoskeleton keratin [67]
*Rn7sk*	4697			

**Figure 6.**
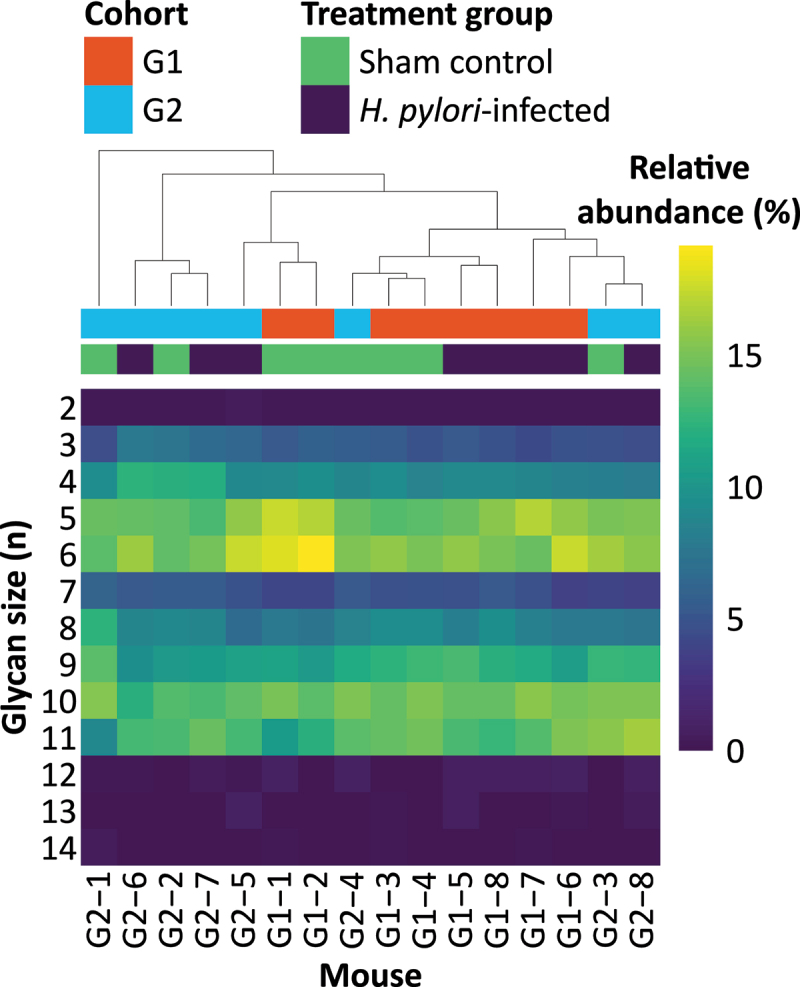
Relative abundance heatmap of glycan sizes. Columns are mice and rows are glycans of different sizes. Size was defined as the number of monosaccharides in the glycan. Each cell shows the pooled relative abundance of all glycans of the same size in that mouse. The dendrogram at the top of the heatmap shows the hierarchical agglomerative clustering (HAC) which was used to cluster the mice. Cohort and treatment denoted by color.

A primary function of SMCs is to secrete mucus containing MUC5AC. Mass spectrometry revealed that the mouse gastric mucin *O*-glycans were large and had a bimodal distribution where glycans of 5–6 or 10–11 monosaccharides in size had the greatest combined relative abundances (Figure 6). In total, 100 glycans were characterized (Supplemental File 4).

**Figure 7.**
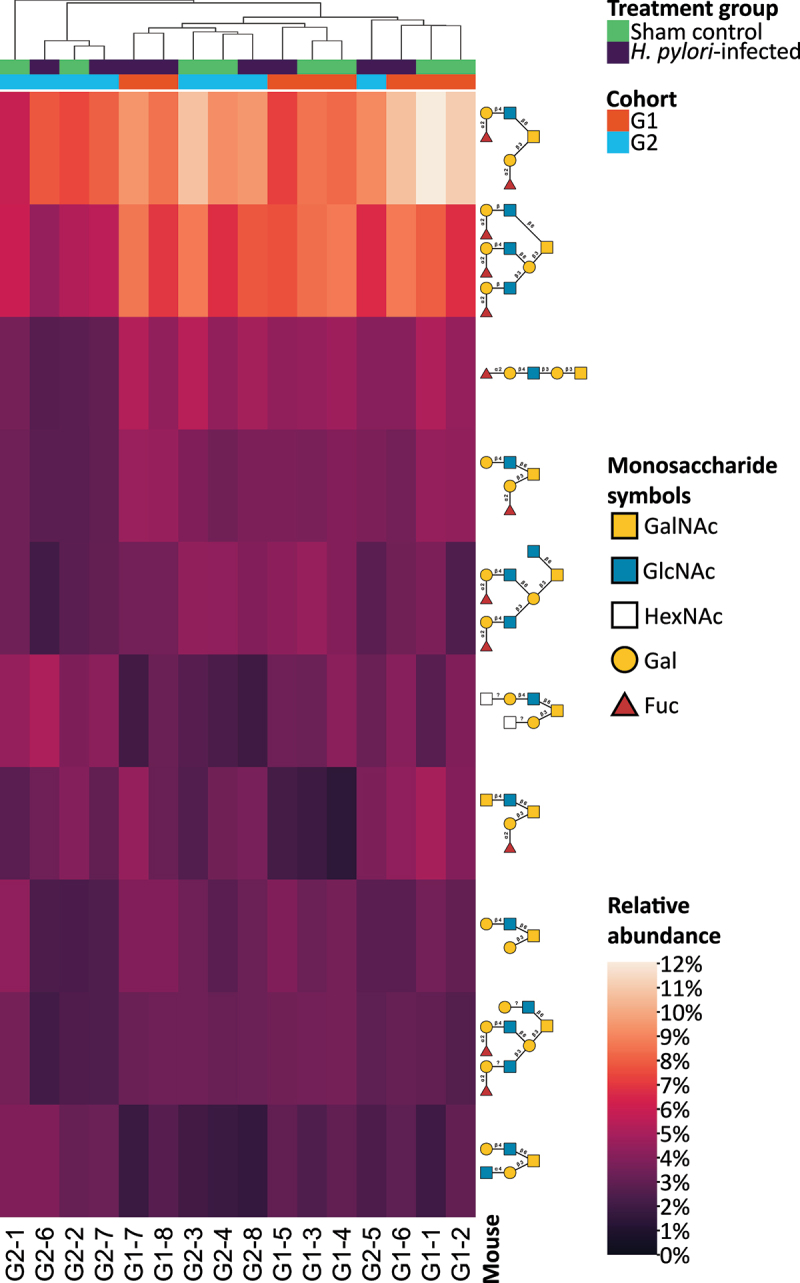
Heatmap of relative abundances of the top 10 most abundant glycans characterized with mass spectrometry. The dendrogram at the top of the heatmap shows the hierarchical agglomerative clustering (HAC) which was used to cluster the mice. Cohort and treatment denoted by color. Glycans were visualized using symbols according to the Symbol Nomenclature for Glycans (SNFG) guidelines. GalNAc = N-Acetylgalactosamine. GlcNAc = N-Acetylglucosamine. HexNAc = Either GalNAc or GlcNAc but it could not be determined which. Gal = Galactose. Fuc = Fucose.

In line with the glycan size distribution, the two most abundant glycans were Fuc(α1–2)Gal(β1–3)[Fuc(α1–2)Gal(β1–4)GlcNAc(β1–6)]GalNAcol and Fuc(α1–2)Gal(β1–3/4)GlcNAc(β1–3)[Fuc(α1–2)Gal(β1–4)GlcNAc(β1–6)]Gal(β1–3)[Fuc(α1–2)Gal(β1–3/4)GlcNAc(β1–6)]GalNAcol, constituting 9 and 7% respectively of the glycan-relative abundance. These two glycans, composed of 6 and 11 monosaccharides respectively, were also representative of another pattern wherein the glycans were heavily fucosylated (Figure 7). The mean relative abundance of fucosylated glycans was 66%. A heatmap of all glycans clustered with hierarchical agglomerative clustering (HAC) is shown in supplemental figure S2. The 10 most abundant glycans made up 43.7% of the total glycan pool and the remaining 90 glycans had mean relative abundances no greater than 2.6%.

The main glycosylation pathway(s) were elucidated by identifying the top five most abundant glycans and mapping all intermediate steps between them and the initial precursor glycan, commonly referred to as Tn [74]. Additional detected glycans were used to fill in steps linking the top five glycans and the Tn antigen, and any remaining gaps were bridged by interpolating presumed intermediary glycans. All these glycans were O-linked glycans. The RNA-Seq data were subsequently mined for glycosyltransferases by manually reviewing detected genes annotated with glycosyltransferase activity (GO term GO:0016757). These genes were ranked by mean expression (TPM) in sham control mice and annotated with supporting evidence from the literature until sufficient glycosyltransferases had been identified to account for all steps in the pathway (s). Ultimately, the 15 most abundant glycosyltransferases with known activity on mucin O-glycans could explain the complete biosynthetic chain of the top five most abundant glycans (Table 2).

**Table 2.** The top 15 most abundant glycosyltransferases with known activity on mucin O-glycans. These glycosyltransferases could explain the biosynthesis of the top 5 most abundant glycans. 12 of these 15 glycosyltransferases could catalyze at least one of the steps required for the glycosylation of these top 5 most abundant glycans.

Gene	Mean TPM	Reaction(s)	Part of glycosylation of top 5 most abundant glycans	Evidence
Gcnt3	294	3 different transfers of GlcNAc to the following: 1: Core 1 GalNac =>Core 2 2: Core 3 GalNac =>Core 4 3: Linear polylactoseamine Gal =>I branch	Yes	Mouse [75]
C1galt1	190	Gal(β1–3) to Tn to create Core 1	Yes	Mouse [76]
B3gnt3	105	GlcNAc(β1–3) to Gal(β1–4)GlcNAc or Gal(β1–3)GlcNAc	Yes	Human [77,78]
Fut2	82	Fuc(α1–2) to terminal Gal	Yes	Mouse [25]
Galnt12	74	GalNac to serine or threonine (“Tn” Glycan)	Yes	Human [79]
Galnt7	60	Tn	Yes	Human [80]
Galnt4	60	Tn	Yes	Mouse [81]
Galnt2	60	Tn	Yes	Human [82]
B4galnt3	54	GalNAc(β1–4) to GlcNAc	No	Human [83]
Fut4	43	Fuc(α1–3) to GlcNAc	No	Mouse [84]
Galnt3	38	Tn	Yes	Mouse [85]
B4galt1	33	Gal(β1–4) to GlcNAc in Gal(β1–4)GlcNAc	Yes	Mouse [86]
Galnt6	26	Tn	Yes	Human [87]
B3gnt2	23	GlcNAc(β1–3) to Gal(β1–4)GlcNAc	No	Mouse [88]
B3galt5	20	Gal(β1–3 to either GlcNAc or GalNAc	Yes	Human [89,90]

**Figure 8.**
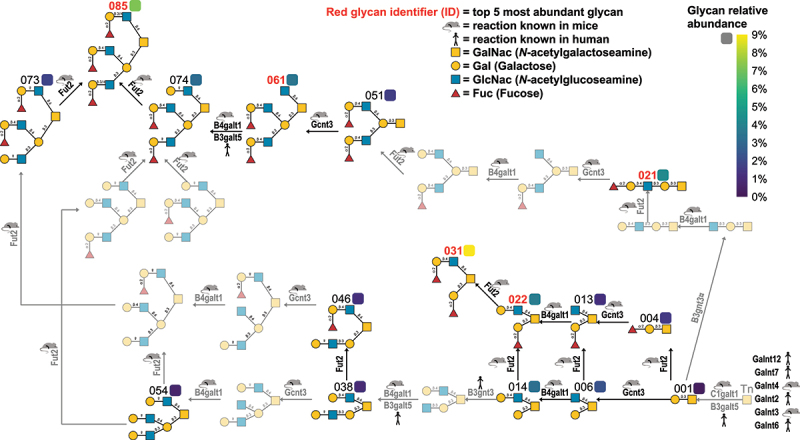
Main glycosylation pathways of surface mucins in the mouse gastric corpus. The top five most abundant glycans are denoted with glycan identifiers (id, see Supplemental File 4) colored red. The color of the boxes next to the id shows the mean relative abundance of that glycan.

The network resulting from mapping the glycosylation of the top five most abundant glycans formed two main pathways ending with either the most abundant glycan 031 (Fuc(α1–2)Gal(β1–3)[Fuc(α1–2)Gal(β1–4)GlcNAc(β1–6)]GalNAcol), or the second most abundant glycan 085 (Fuc(α1–2)Gal(β1–3/4)GlcNAc(β1–3)[Fuc(α1–2)Gal(β1–4)GlcNAc(β1–6)]Gal(β1–3)[Fuc(α1–2)Gal(β1–3/4)GlcNAc(β1–6)]GalNAcol). Glycan 085 was capped with three H antigen (Fuc(α1–2)Gal (β)GlcNAc) terminal motifs, while glycan 031 was capped with one H antigen terminal motif. See Figure 8 for a diagram of the main glycosylation pathways. Spectra annotation of diagnostic ions for glycan 085 is shown in Supplemental Figure S3.

The pathways were charted by identifying glycosylation steps between them and the initial “Tn” glycan (GalNAcol). Intermediate glycans which were identified by mass spectrometry have ID with black text. The transparent glycans are intermediate glycans which were not identified with mass spectrometry but rather are inferred, for example, the Tn glycan which is the precursor to all other O-linked glycans on mucins. The most abundant glycans, 031 and 085, form the end of the main glycosylation pathways.

Glycosyltransferases conducting these steps were identified by datamining the RNA-Seq data to identify the most abundant glycosyltransferases, and then which of these were known to catalyze the required reactions to connect the identified glycans. Glycosyltransferase activity evidence comes directly from experiments on mice (denoted with mouse symbol), or is inferred based on homology to human proteins where such a reaction is known (denoted with stick figure symbol). Glycans were visualized using symbols according to the SNFG guidelines.

### Mucin glycosylation did not change with H. pylori infection

**Figure 9.**
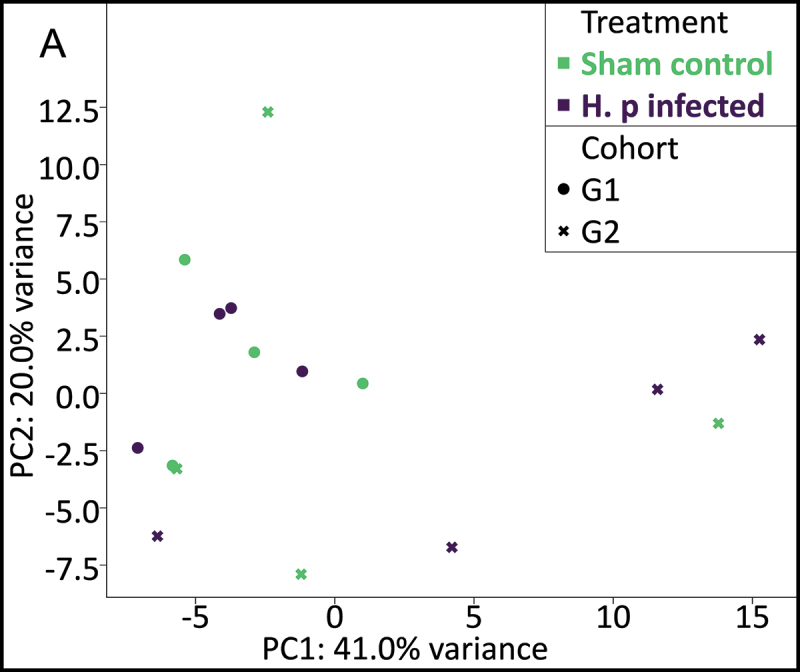
Principal component analysis (PCA) of terminal motif relative abundances using glycowork. Terminal structures of one to three monosaccharides in size were selected as features. 61% of the variance was captured principal components one (PC1) and two (PC2) together. Which cohort the mouse belonged to is denoted with shape (dot or cross), while the treatment that the mouse received is denoted by color.

Glycomics analysis was deepened in glycowork by analysing the glycan motif level, based on terminal motifs that were one to three monosaccharides in size. PCA did not cluster according to any discernible pattern, including treatment and cohort (Figure 9).

**Figure 10.**
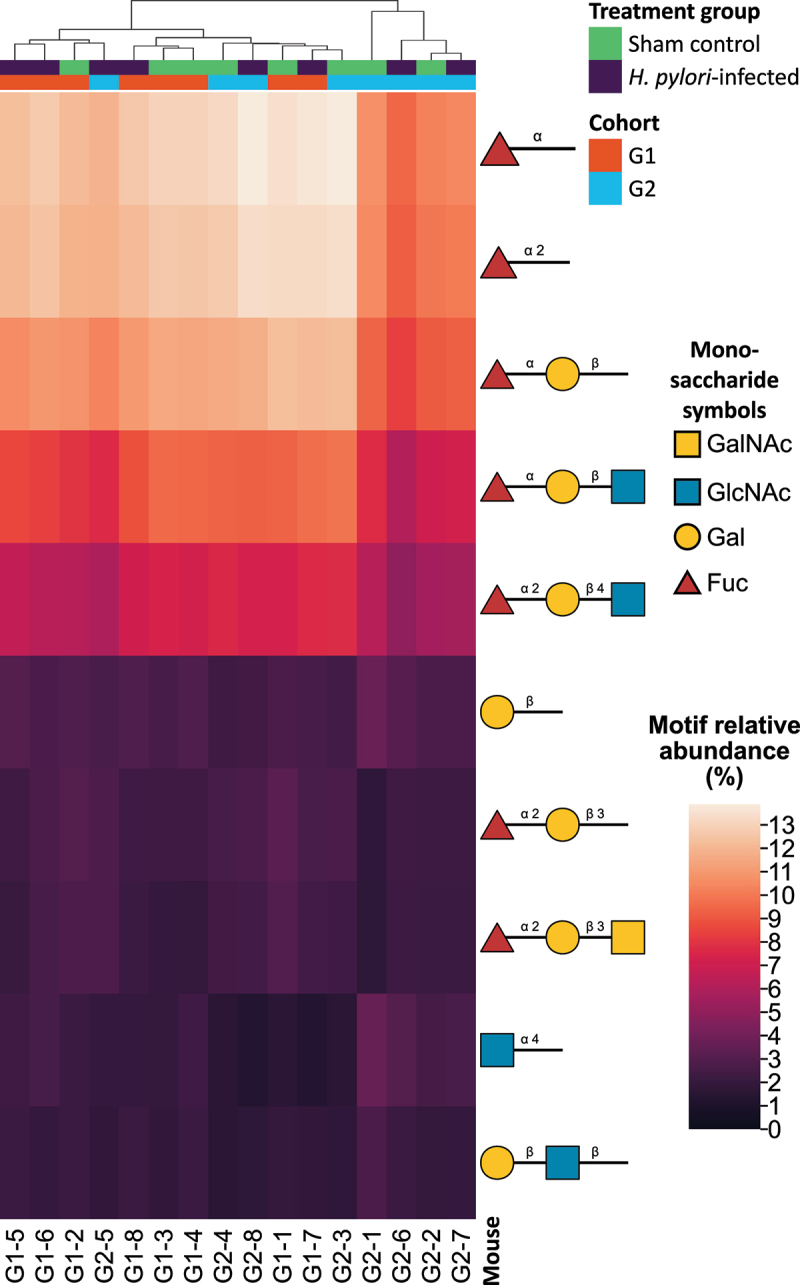
Heatmap of relative abundances of the top 10 most abundant terminal glycan motifs. The complete heatmap with all terminal motifs are shown in Supplemental Figure S4. Terminal motifs of one, two, or three monosaccharides in size were identified using the python package glycowork. The dendrogram at the top of the heatmap shows the hierarchical agglomerative clustering (HAC) which was used to cluster the mice. Cohort and treatment are denoted by color. Glycans are visualized using symbols according to the SNFG guidelines. GalNAc = N-Acetylgalactosamine. GlcNAc = N-Acetylglucosamine. Gal = Galactose. Fuc = Fucose.

Likewise, HAC based on a heatmap of these terminal motifs did not demonstrate any clustering based on treatment or cohort (Figure 10). The heatmap also revealed that the relative abundances of fucosylated terminal motifs dwarfed the relative abundances of non-fucosylated motifs. A small cluster of four mice containing two infected and two sham control (the cluster on the right) contained a few percent less fucosylated terminal motifs. A large proportion of the terminal motifs were H antigens, primarily H type 2 (Fuc(α1–2)Gal(β1–4)GlcNAc). Glycans with at least one H-type antigen constituted 49.7% of the relative abundance. Among the 10 most abundant terminal epitopes was α1,4GlcNAc, which has been shown to inhibit *H. pylori* growth [91]. Sialylated glycans were rare, with Neu5Ac and Neu5Gc moieties constituting 1.6 % and 0.63 % of the relative abundance respectively, while sulfated glycans were even more rare at 0.098 % of the relative abundance.

Differential glycomics in glycowork yielded zero terminal motifs that had different relative abundances between sham control and *H. pylori*-infected mice (Supplemental File 5). There were significant differences in relative abundances of terminal motifs between the two cohorts, although they were small with no log2 fold changes (l2fc) above ±2 and only one l2fc above ±1 (Supplemental Figure S4 and Supplemental File 6).

### Early H. pylori infection changes the gene expression of mouse SMCs

The differential gene expression was first assessed with the unsupervised machine learning methods PCA and UMAP, to determine if samples clustered together based on infection status.

**Figure 11.**
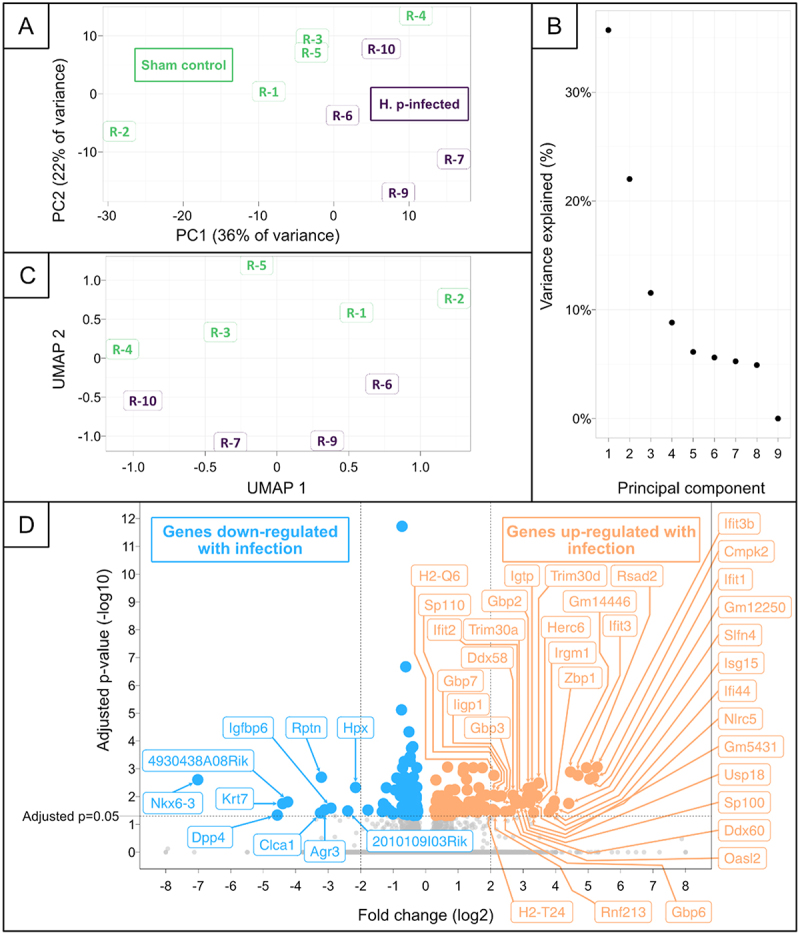
Differential gene expression between infected and sham control mice, as determined with DESeq2 and Independent Hypothesis Weighting (IHW). A: PCA of regularized log transformed gene expression data. Data from H. pylori infected mice was colored purple while data from sham control mice was colored green. PC1 and PC2 together captured 58% of the variance. B: Dotplot visualizing the proportion of variance captured by each principal component from the PCA in panel A. The captured variance plateaued after PC5. C: UMAP dimensionality reduction of principal components 1 through 5 from the PCA in panel A. D: Volcano plot of differential gene expression results from DESeq2 after adjusting with IHW. Significant genes (adjusted *p*-value < 0.05) are above the horizontal dotted line. Genes which are down-regulated with H. pylori infection are to the left and are colored blue, while genes which are up-regulated with H. pylori infection are to the right and are colored orange. Furthermore, genes with a log2 Fold change less than −2 or greater than 2 are highlighted with their respective gene symbols in text boxes.

PCA and subsequent UMAP dimensionality reduction of principal components 1 through 5 showed that infected and sham control mice clustered separately, but with some overlap (Figure 11(A and C)). Principal components for UMAP dimensionality reduction were chosen based on a plateauing of captured variance in subsequent principal components (Figure 11B). In the PCA, *H. pylori*-infected mice tended to have greater values of PC1 and lower values of PC2 as compared to the sham control mice (Figure 11A). Meanwhile, *H. pylori*-infected mice tended to have lower UMAP 2 values than the sham control mice, while no differences between the two groups was observed for UMAP 1 (Figure 11C).

Differentially-expressed genes between infected and sham control mice were identified using DeSeq2 and IHW. Of the 23,678 genes detected in the tissue (Supplemental File 7), 214 showed significant differential expression (adjusted *p*-value < 0.05, Figure 11D, and Supplemental File 8). In *H. pylori*-infected mice, 33 of the significant genes were up-regulated with a log2 fold change greater than 2, while ten were down-regulated with a log2 fold change less than −2. None of the very abundantly-expressed genes in normal SMCs (Table 1) were differentially expressed.

### Gene set enrichment analysis indicated a broad perturbation of genes related to oxidative phosphorylation and infection

**Figure 12.**
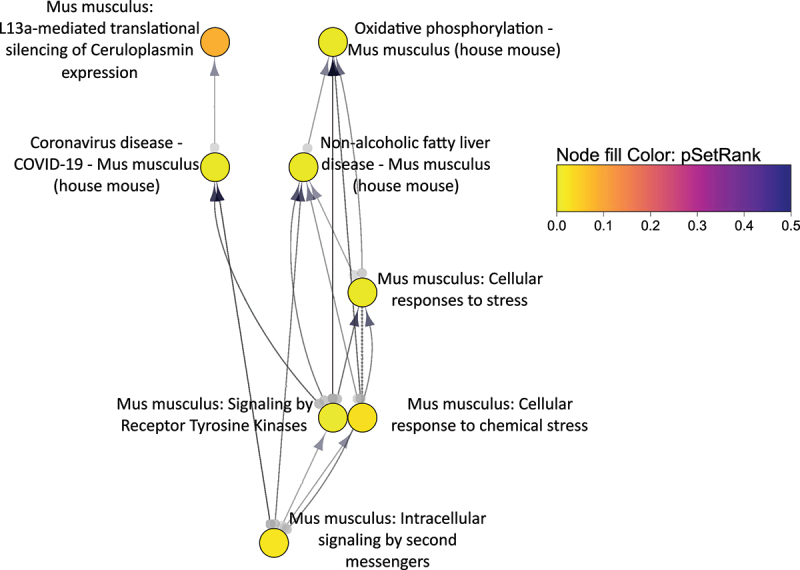
Cytoscape visualization with hierarchical layout of significant gene sets from SetRank network. The R package SetRank output a network of enriched gene sets. Gene sets with pSetrank < 0.05 were selected and analysed in Cytoscape through the R package RCy3. The network style was a modification of the SetRank style provided by the R package. The directed edges indicate that the gene sets overlap and the arrow points towards which of the two overlapping gene sets that contained a greater proportion of higher ranked genes. The hierarchical layout was applied with default settings, which arranged the nodes to avoid edge crossings and consider the directionality of the edges. The topmost nodes of the hierarchical layout were Oxidative phosphorylation and L13a-mediated translational silencing of Ceruloplasmin expression, which had incoming edges but no outgoing edges. The Ceruloplasmin gene itself (Cp) was not differentially-expressed.

The ranked gene set enrichment analysis with SetRank yielded 119 significant gene sets (adjusted *p*-value < 0.05, Supplemental File 9). To narrow down the significant gene sets to a more manageable number for analysis we applied a filter of the variable pSetRank (<0.05) and analysed the resulting network of 8 gene sets in Cytoscape using RCy3 (Figure 12). A hierarchical layout was applied which showed that highly ranked genes in the two gene sets “Oxidative phosphorylation” (from KEGG) and “L13a-mediated translational silencing of Ceruloplasmin expression” (from Reactome) drives at least part of the significance [46] of the other 6 gene sets “non-alcoholic fatty liver disease,” “coronavirus disease – COVID-19,” “cellular responses to stress,” “cellular response to chemical stress,” “signalling by receptor tyrosine kinases,” and “intracellular signaling by second messengers.”

The Ceruloplasmin gene (*Cp*) itself, from the gene set bearing its name, did not show significant differential expression. This gene set corresponds to a Reactome pathway and was created on the basis of a study describing how interferon gamma (IFN-γ) silences *Cp* [92]; therefore, other components of this gene set are likely perturbed without directly suppressing *Cp* expression.

### Up-regulated genes were often inducible by interferon type I, ii, or iii, and were mostly co-expressed in a functionally related cluster

**Figure 13.**
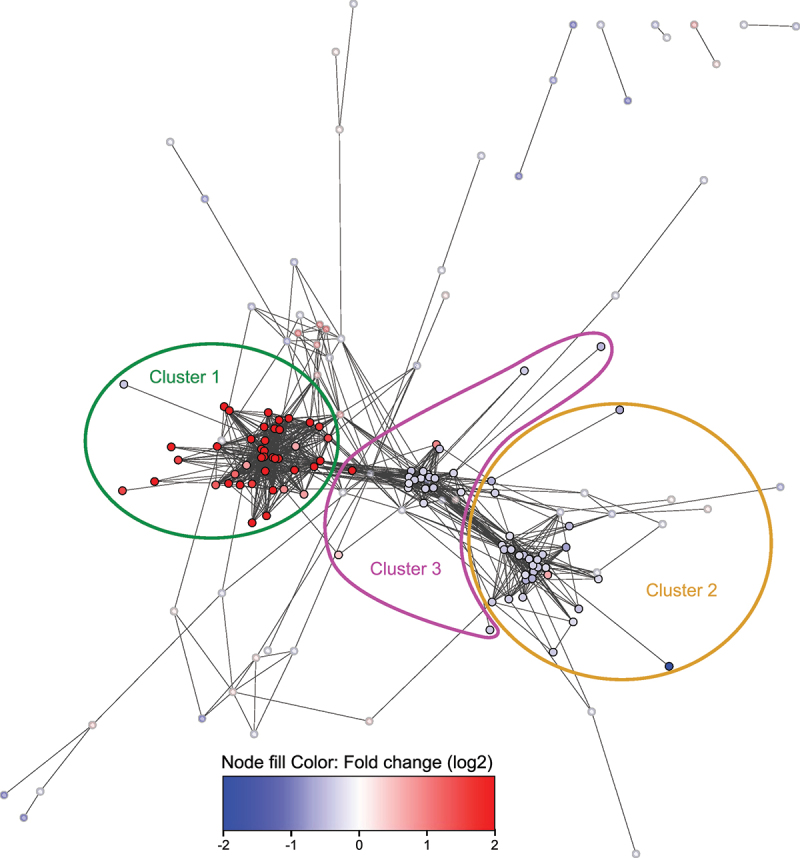
Cytoscape string network of significantly differentially-expressed genes. R packages were used to create a cytoscape network based on full string interactions with default confidence score of 400. Significant genes without a string interaction to another significant gene were not included in the network. Node fill was colored by log2 Fold changes on a continuous scale from blue (down-regulated) to red (up-regulated) with H. pylori infection. Minimum and maximum values in the color scale was set to − 2 and 2 respectively to facilitate visualization, where the more extreme Fold-changes would otherwise distort the scale. There were 3 visually obvious clusters, and they were picked out with a systematic approach using MCL clustering with the default inflation parameter of 2.5 and selecting the top 3 clusters. To highlight these 3 clusters, the opacity of all nodes which did not belong to them was reduced. The colored loops indicate which cluster the full opacity nodes within belonged to. The supplemental file 1 cytoscape file contains further details, such as which genes the nodes consist of.

By visualizing the interactions from the STRING database of all significant genes, 3 distinct clusters emerged (Figure 13). Most up-regulated genes in the network were present in cluster 1, while the other two clusters consisted mostly of slightly down-regulated genes. A large version of Figure 13 with full annotation of all differentially-expressed genes mapped to the STRING database is available in the supplementary (Supplemental Figure S5).

A large proportion of all significantly up-regulated genes was located in cluster 1 (41 of 90 genes; 46%), particularly among the most strongly up-regulated genes with a log2 fold change > 2 (27 of 33 genes; 82%). All up-regulated genes in cluster 1 were either interferon-stimulated genes (ISGs) inducible by at least one interferon type (I, II, or III) or capable of inducing interferon production themselves (Table 3). This aligned with the observation that one of the two significantly enriched gene sets from the SetRank analysis, “L13a-mediated translational silencing of Ceruloplasmin expression” (Figure 12), is related to IFN-γ (type II interferon) signaling [92].

**Table 3.** Genes which constituted cluster 1 from Figure 13. 41 of the 42 genes in the cluster were up-regulated. All 41 up-regulated genes in cluster 1 were either inducible by at least one type of interferon (type I, type II, or type III), or induced interferon production itself.

Gene	log2 Fold Change	Type I interferon induced gene	Type II interferon induced gene	Type III interferon induced gene	Effect on type I interferon production
Bst2	1,57	Yes [93]	Yes [94]		
Cmpk2	5,17	Yes [95]	Yes [96]	Yes [97]	
Ddx58	2,69				Stimulates [98,99]
Ddx60	2,79	Yes [100]			
Eif2ak2	0,76	Yes [98]			
Gbp2	3,15	Yes [97]	Yes [101,102]	Yes [97]	
Gbp3	2,76	Yes [97]	Yes [101,102]	Yes [97]	
Gbp5	1,86		Yes [101,103]		
Gbp6	2,45	Yes [97]	Yes [101,102]	Yes [97]	
Gm14446	4,46	Yes [104]			
Helz2	0,89	Yes [105]			
Herc6	3,43	Yes [106]			
Ifi27	0,67	Yes [107]			
Ifi44	3,79	Yes [108]		Yes [97]	
Ifit1	5,05	Yes [99]		Yes [97]	
Ifit2	2,82	Yes [99]			
Ifit3	4,69	Yes [99]	Yes [109]	Yes [97]	
Ifit3b	5,28	Yes [104]			
Igtp	3,27		Yes [110]		
Iigp1	2,33	Yes [97]	Yes [111]	Yes [97]	
Irgm1	3,23	Yes [97]	Yes [112,113]	Yes [97]	
Isg15	3,86	Yes [98]		Yes [97]	
Lgals3bp	0,74	Yes [114]	Yes [114]		
Nlrc5	3,21		Yes [115]		Inhibits [115]
Oasl2	2,61	Yes [97]	Yes [116]	Yes [97]	
Parp14	1,86	Yes [97]	Yes [117]	Yes [97]	
Phf11d	1,82	Yes [97]		Yes [97]	
Psmb8	1,64	Yes [118]			
Psmb9	1,46	Yes [119]		Yes [97]	
Rnf213	2,14	Yes [120]	Yes [120]		
Rsad2	4,95	Yes [99]	Yes [108]	Yes [97]	
Slfn4	3,93	Yes [121]			
Sp100	2,82	Yes [99]	Yes [122]	Yes [97]	
Sp110	2,10	Yes [97]		Yes [97]	
Tlr3	1,20		Yes [123]		Stimulates [124]
Tnfsf10	1,50	Yes [97]	Yes [125]	Yes [97]	
Tpt1	−0,44				
Trim14	1,48	Yes [109]	Yes [109]		
Trim30a	3,14	Yes [97]		Yes [97]	
Trim30d	3,48	Yes [97]		Yes [97]	
Usp18	2,98	Yes [97]		Yes [97]	
Znfx1	1,28	Yes [126]			

### Genes involved in oxidative phosphorylation are down-regulated in mouse SMCs infected with H. pylori

Cluster 2 consisted of 29 down-regulated genes and one up-regulated gene. When investigating these 30 genes, we discovered that they largely are related to OXPHOS (Table 4). OXPHOS was also one of the top two most important gene sets according to the SetRank analysis (Figure 12). Thus, we conclude that *H. pylori* infection leads to a down-regulation of OXPHOS in SMCs in vivo.

**Table 4.** Genes which formed cluster 2 (Figure 13). All genes except mt-Nd6 were down-regulated in this cluster. With few exceptions, these genes were either components of oxidative phosphorylation or directly affected this process in some other way.

Gene	log2FoldChange	Component of OXPHOS complex	Other direct effect on OXPHOS
2010107E04Rik	−0.73	Complex V	
2310036O22Rik	−0.40		
4930438A08Rik	−4.25	Complex V	
Atp5e	−0.37	Complex V	
Atp5h	−0.44	Complex V	
Atp5j	−0.48	Complex V	
Atp5j2	−0.30	Complex V	
Atp5o	−0.28	Complex V	
Atp6v1d	−0.30		
Chchd10	−0.42		Required for complex I assembly [127]
Chchd2	−0.41		Required for complex I assembly [127]
Cox4i1	−0.32	Complex IV	
Cox6a1	−0.40	Complex IV	
Cox6c	−0.61	Complex IV	
Cox7b	−0.40	Complex IV	
Gm3244	−0.61	Complex I	
Minos1	−0.29		Important for Cristae formation [128]
Mrpl43	−0.39		Component of mitochondrial ribosomes [129]
Mrps6	−0.61		Component of mitochondrial ribosomes [129]
mt-Nd6	0.66	Complex I	
Ndufa13	−0.51	Complex I	
Ndufa6	−0.45	Complex I	
Ndufb10	−0.29	Complex I	
Ndufs4	−0.31	Complex I	
Ndufs6	−0.41	Complex I	
Pet100	−0.49		Important for the assembly of oxidative phosphorylation complexes [130]
Ppif	−0.55		Modulates complex V [131]
Slc5a3	−0.66		
Tomm7	−0.42		Component of mitochondrial protein import TOM complex [132]
Uqcrq	−0.36	Complex III	

### H. pylori infection down-regulates genes related to protein synthesis which are connected to OXPHOS and ISGs

The 22 genes in cluster 3 were primarily associated with ribosomes and protein synthesis (Table 5). All genes encoding ribosomal subunits within this cluster also belonged to one of the two most important gene sets identified by the SetRank analysis, namely “L13a-mediated translational silencing of Ceruloplasmin expression” (Figure 12). All but three genes in cluster 3 were down-regulated; among the up-regulated genes, *Zbp1* showed a notably high log2 fold change of 3.96. Like other up-regulated genes in cluster 1, Zbp1 is linked to interferon stimulation [137].

**Table 5.** Genes from cluster 3 (Figure 13). All genes in this cluster, except Srp54a, Xrn2, and Zbp1, were down-regulated. Most genes encoded ribosomal subunits or influenced protein synthesis through other mechanisms. All ribosomal genes in cluster 3 also belonged to the gene set “L13a-mediated translational silencing of Ceruloplasmin expression,” which was one of the two most important perturbed gene sets according to the SetRank analysis (Figure 12).

Gene symbol	log2FoldChange	Component of ribosomes	Part of gene set “L13a-mediated translational silencing of Ceruloplasmin expression”	Other relationships to protein synthesis
*Abtb1*	−0.56			Probable elongation factor protein [52]
*Btf3*	−0.32			Translational regulation, potentially protection against intracellular bacteria [133]
*Eef1b2*	−0.35			Probable elongation factor protein [52]
*Fkbp2*	−0.40			Probable PPIase which facilitates protein folding [52]
*Pdcd5*	−0.31			Induces apoptosis [134]
*Rpl13a*	−0.47	60S ribosomal subunit protein [52]	Yes	
*Rpl18*	−0.34	60S ribosomal subunit protein [52]	Yes	
*Rpl19*	−0.48	60S ribosomal subunit protein [52]	Yes	
*Rpl23a*	−0.35	60S ribosomal subunit protein [52]	Yes	
*Rpl29*	−0.37	60S ribosomal subunit protein [52]	Yes	
*Rpl34*	−0.39	60S ribosomal subunit protein [52]	Yes	
*Rplp0*	−0.40	60S ribosomal subunit protein [52]	Yes	
*Rplp2*	−0.38	60S ribosomal subunit protein [52]	Yes	
*Rps11*	−0.42	40S ribosomal subunit protein [52]	Yes	
*Rps26*	−0.28	40S ribosomal subunit protein [52]	Yes	
*Rps3a1*	−0.26	40S ribosomal subunit protein [52]	Yes	
*Rps9*	−0.38	40S ribosomal subunit protein [52]	Yes	
*Srp54a*	0.93			Signal recognition particle (SRP) complex subunit [52]
*Tubb4b*	−0.42			Might affect ribosomes through its association with RNA granules [135]
*Xrn2*	0.42			Exoribonuclease which may stop mRNA transcription [136]
*Zbp1*	3.96			Mediates type I interferon responses from DNA sensing, thus suppresses global protein translation [137]
*Zfp622*	−0.48			Helps assemble ribosomal 60S subunit [138]

**Figure 14.**
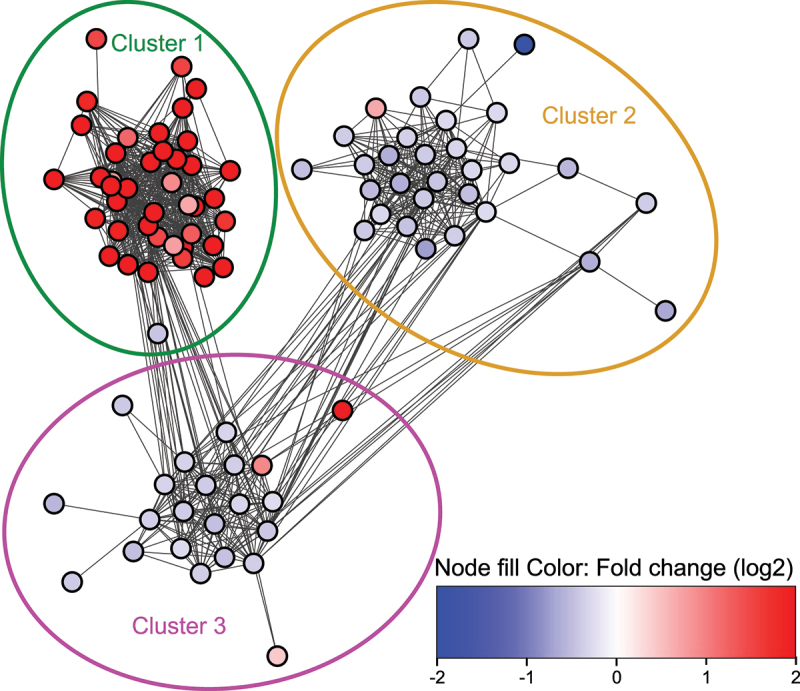
The main clusters 1–3 were highly interconnected. There was a high degree of string interaction interconnectivity between cluster 1 and 3, and between cluster 2 and 3. There were no direct interactions between cluster 1 and 2. The cytoscape file contains further details, such as which genes the nodes consist of (supplemental file 1).

By drawing a sub-network of the genes belonging to clusters 1–3 the interconnectivity between these 3 clusters was visualized (Figure 14). Here, it was evident that cluster 3 had many connections with both the up-regulated interferon-inducible genes of cluster 1 and the down-regulated genes related to oxidative phosphorylation of cluster 2, while there were no direct interactions between clusters 1 and 2.

### Inflammation-related non-coding miRNA Gm23935 is the most abundant differentially-expressed gene

**Figure 15.**
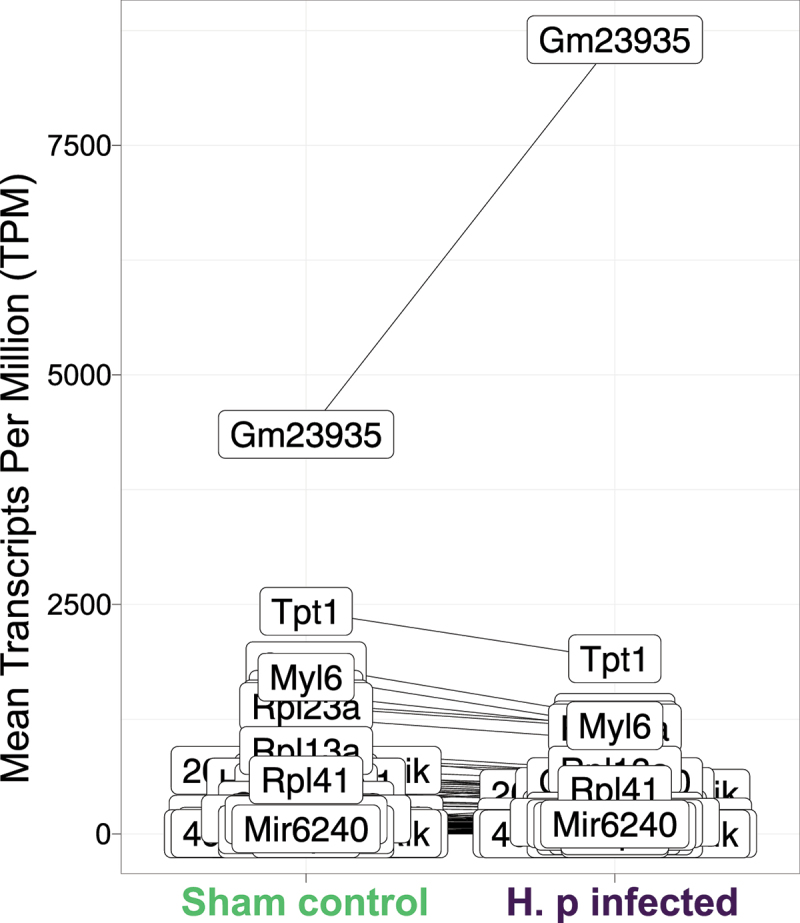
Significantly differentially-expressed genes mean TPM (Transcripts per Million) of infected vs sham control mice. The most highly-expressed gene with a significant differential gene expression was the non-coding miRNA Gm23935. Gm23935 was up-regulated in H. pylori infected mice and had a modest log2 Fold change of 0.9.

Highly-expressed genes might be important, despite a small log2 fold change. To discover such important genes, we investigated the mean TPM of all differentially-expressed genes in the two experimental groups (Figure 15). The non-coding miRNA *Gm23935* had the greatest overall gene expression. In contrast, the log2 fold change of *Gm23935* was modest at 0.9. Gm23935 is known to be enriched in NLR family pyrin domain containing 3 (NLRP3) inflammasomes [139], however, *Nlrp3* was not differentially-expressed in our data set. *Gm23935* is also differentially-expressed in hypo-perfused mouse brain cells [140]. Thus, the most highly-expressed, differentially-expressed gene appears associated with inflammation.

### Nkx6-3 had the most extreme differential expression with a log2 Fold change of −7, and depletion of this gene is a known contributor to gastric cancer development

43 significantly differentially-expressed genes had an absolute log2FoldChange (l2fc) of >|2| (Table 6). Out of the 33 genes in Table 6 with a l2fc > 2 (up-regulated in *H. pylori*-infected mice), 32 genes were known to be inducible by interferons. Among the down-regulated genes with l2fc < −2, one gene was known to be essential for mucin expansion (*Clca1*) [141]. Finally, *Nkx6-3*, coding for a homeobox protein related to gastric cell differentiation and tumor suppression, had the most extreme log2 fold change (−7). Notably, the depletion of this protein has been shown to contribute to gastric cancer development [146,147].

**Table 6.** Top significantly differentially-expressed genes based on analysis with Deseq2 and IHW. The table shows which of the differentially-expressed genes (adjusted *p*-value < 0.05) that had a log2FoldChange > 2 or <−2. The up-regulated genes in the table were genes whose overall expression level could be associated with inflammation.

Gene symbol	log2FoldChange	Relevance to previous analyses
*Nkx6-3*	−7.02	Not in cluster 1–3.
*Dpp4*	−4.57	Not in cluster 1–3.
*Krt7*	−4.40	Not in cluster 1–3, but is a type II keratin [52] just like the highly-expressed gene Krt8 (Table 1).
*4930438A08Rik*	−4.25	Codes for an OXPHOS complex V subunit cluster 2 (Table 4).
*Clca1*	−3.24	Not in cluster 1–3, but is essential for mucus expansion [141].
*Rptn*	−3.21	Not in cluster 1–3
*Agr3*	−3.11	Not in cluster 1–3
*Igfbp6*	−2.92	Not in cluster 1–3, but is a secreted protein [52].
*2010109I03Rik*	−2.40	Not in cluster 1–3. Also known as *Ly6m* [52].
*Hpx*	−2.16	Not in cluster 1–3
*H2-T24*	2.07	Not in cluster 1–3, but codes for a histocompatibility protein [52] which interacts with Usp18 in cluster 1 (Figure 13). Inducible by interferon type I [142].
*Sp110*	2.10	In cluster 1, induced by interferon type 1/3 (Table 3).
*H2-Q6*	2.10	Not in cluster 1–3, but codes for a histocompatibility protein [52] which interacts indirectly with cluster 1 through H2-T24, H2-Q4, Tap2, and B2m (Figure 13). Inducible by interferon type I [143] and type II [144].
*Rnf213*	2.14	In cluster 1, induced by interferon type 1/2 (Table 3).
*Gbp7*	2.22	Not in cluster 1–3, but codes for an interferon-inducible GTPase that protects against bacterial infections [101].
*Iigp1*	2.33	In cluster 1, induced by interferon type 1/2/3 (Table 3). Also known as *Irga6* [52].
*Gbp6*	2.45	In cluster 1, induced by interferon type 1/2/3 (Table 3).
*Oasl2*	2.61	In cluster 1, induced by interferon type 1/2/3 (Table 3).
*Ddx58*	2.69	In cluster 1, stimulates type I interferon production (Table 3). Also known as *Rigi* [52].
*Gbp3*	2.76	In cluster 1, induced by interferon type 1/2/3 (Table 3).
*Ddx60*	2.79	In cluster 1, induced by interferon type 1 (Table 3).
*Ifit2*	2.82	In cluster 1, induced by interferon type 1 (Table 3).
*Sp100*	2.82	In cluster 1, induced by interferon type 1/2/3 (Table 3).
*Usp18*	2.98	In cluster 1, induced by interferon type 1/3 (Table 3).
*Trim30a*	3.14	In cluster 1, induced by interferon type 1/3 (Table 3).
*Gbp2*	3.15	In cluster 1, induced by interferon type 1/2/3 (Table 3).
*Nlrc5*	3.21	In cluster 1, induced by interferon type 2, and inhibits production of interferon type I (Table 3).
*Irgm1*	3.23	In cluster 1, induced by interferon type 1/2/3 (Table 3).
*Igtp*	3.27	In cluster 1, induced by interferon type 2 (Table 3).
*Gm5431*	3.32	Not in cluster 1–3, but has predicted coding for a GTP-binding protein which is predicted to respond to interferon-beta stimulation [52].
*Herc6*	3.43	In cluster 1, induced by interferon type 1 (Table 3).
*Trim30d*	3.48	In cluster 1, induced by interferon type 1/3 (Table 3).
*Ifi44*	3.79	In cluster 1, induced by interferon type 1/3 (Table 3).
*Isg15*	3.86	In cluster 1, induced by interferon type 1/3 (Table 3).
*Slfn4*	3.93	In cluster 1, induced by interferon type 1 (Table 3).
*Zbp1*	3.96	In cluster 3, induced by IFN type I stimulation (Table 5).
*Gm12250*	4.40	Not in cluster 1–3, but is translated to IRB10 which is induced by type 1 interferon [145].
*Gm14446*	4.46	In cluster 1, induced by interferon type 1 (Table 3).
*Ifit3*	4.69	In cluster 1, induced by interferon type 1/2/3 (Table 3).
*Rsad2*	4.95	In cluster 1, induced by interferon type 1/2/3 (Table 3).
*Ifit1*	5.05	In cluster 1, induced by interferon type 1/2 (Table 3).
*Cmpk2*	5.17	In cluster 1, induced by interferon type 1/2/3 (Table 3).
*Ifit3b*	5.28	In cluster 1, induced by interferon type 1 (Table 3).

### Lgals3bp (cluster 1) and Rpl19 (cluster 2), but not Cox6c (cluster 3) gene expression was validated at the protein level through immunofluorescence (IF)

To validate the overall RNA-level results, we selected one protein-coding gene from each cluster 1–3. Protein choice was based on high gene expression and the availability of adequately verified antibodies on mouse tissue. LGALS3BP (cluster 1), also known as Tumor-associated antigen 90K [52], is a type I and II interferon induced gene [114]. In humans, LGALS3BP has been implicated in the immune defense against cancer and pathogens [148]. RPL19 (cluster 2) is a component of the large ribosomal subunit [52]. The mitochondrial protein COX6C (cluster 3) is a cytochrome c oxidase (complex IV) subunit [52]. TNFα stimulation and *C. rodentium* infection decreases COX6C expression in the mouse colon, and this reduction is reversed with IL-4 and IL-6 treatment [149].

**Figure 16.**
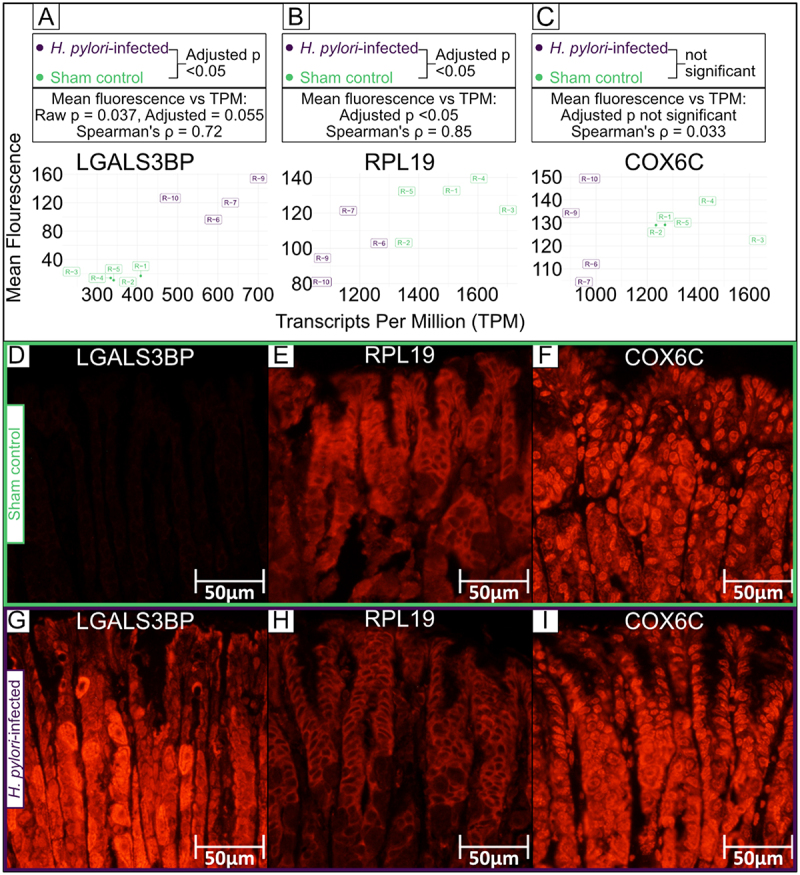
Immunofluorescence protein-level validation of protein coding genes belonging to RNA-Seq clusters 1–3.A-C: Mean fluorescence of SMCs correlated to the gene expression (TPM) of the gene coding for the protein. D-I: Representative IF images (40x magnification) from sham control (D-F) and H. pylori-infected mice (G-I). Cluster 1 (a, d, G) antibody recognizing RPL19. Cluster 2 (b, e, H) antibody recognizing LGALS3BP Cluster 3 (C, F, I) antibody recognizing COX6C.

Mean signal intensity of LGALS3BP (Figure 16A, D, and G) and RPL19 (Figure 16B, E, and H) differed significantly between *H. pylori*-infected and sham control mice, while COX6C (Figure 16C, F, and I) did not. Similarly, there was a significant positive correlation between LGALS3BP signal intensity and *Lgals3bp* gene expression, but not between COX6C signal intensity and *Cox6c* gene expression. The raw p value for the positive correlation (Spearman’s ρ = 0.72) between RPL19 signal intensity and *Rpla19* gene expression was 0.037, but when adjusted for multiple comparisons it was bordering to significant (adjusted *p* = 0.055). IF Images and associated code are available in Supplemental File 11.

## Discussion

Here, we demonstrated that the collection of the SMCs using LCM yielded sufficient RNA quantity and quality for RNA-Seq in 9/10 mice, without the need for amplification prior to library preparation. Genes coding for secreted mucus proteins, mitochondrial function, and cellular structure were hallmarks of the normal SMC. During early *H. pylori* infection, 214 genes were differentially-expressed, while 119 Reactome and KEGG gene-sets were perturbed. Network analyses of significant genes and gene-sets showed that *H. pylori* infection causes an up-regulation of interferon-stimulated genes (ISGs), a down-regulation of both oxidative phosphorylation (OXPHOS) and protein synthesis-related genes, and the initiation of an early pre-cancerous cascade with a depletion of *Nkx6-3*.Furthermore, glycan analysis using mass spectrometry expanded the known mouse gastric glycome and revealed two main glycosylation pathways characterized by large and heavily fucosylated glycans, and this was corroborated by glycosyltransferase expression. Finally, the mouse gastric glycome was remarkably unchanged by early *H. pylori* infection.

### The normal mouse SMC of the gastric corpus contains abundant RNA coding for tumor suppressors, secreted proteins, components of cellular respiration, and structural proteins

The gene expression of normal (sham control) SMCs was dominated by a high expression of well-known marker genes *Muc5Ac*, *Tff1*, *Gkn1*, *Gkn2*, *Psca*, and *Lgals2* [17,54]. The proteins coded by these genes can all be secreted into mucus. Of these, *Tff1*, *Gkn1*, and *Gkn2* are also known as tumor suppressor genes [62,64,150]. Another highly-expressed, tumor-suppressing gene was *Mal* [69].

Although present in the data, a notable absence among the highly-expressed genes was *Il33*, which was a canonical marker gene in both PanglaoDB [54] and Bockerstett 2020 [17]. In Bockerstett 2020, which used Balb mice, there were two different clusters annotated as SMCs, cluster 0 and cluster 2, and healthy cells were part of both clusters. *Il33* defined cluster 0 together with *Tff1* and *Muc5ac*, but not cluster 2, which was only defined by *Tff1* and *Muc5ac*. In humans, according to the protein atlas, the expression of *Il33* is more concentrated in the isthmus, where the isthmus stem cells (IsthSCs) reside [151], then decreasing below detection limit toward the surface [152]. Thus, *Il33* might be associated with less differentiated SMCs, while fully differentiated SMCs reduce or stop expressing *Il33*. Similarly, in C57BL/6 mice, *Il33* was expressed in the isthmus SMCs as well as the mucus neck cells, but not higher up in the pits or on the surface [153].

*Il33 was* not differentially expressed in the present study, however, in Balb mice infected with an 11 times higher dose of *H. pylori* strain 26,695, there was an increase of IL-33 and its receptor ST-2 in SMCs and other cells 2 weeks after infection [154]. The authors did not quantify the inflammation but described it as severe, and the H&E micrographs provided appeared more inflamed than in the present study. If *Il33* decreases with SMC differentiation, then one potential mechanism behind the differential expression could be reduced lifespan of the SMCs. *In vitro* results from the same article showed that the IL-33 could be both unchanged, up-regulated, and down-regulated depending on infection time and dose with *H. pylori*, which might explain why *Il33* and *Il1rl1* (which codes for ST-2) was neither highly nor differentially expressed in the present study’s data. Different *H. pylori* and mouse strains might also explain differences in results.

In humans, FCGBP is expressed in SMCs and forms a heteromer with TFF1 [62]. While *Fcgbp* was detected in the present mouse data, it was of very low abundance, indicating that another protein might fill the same role in mice. Another gene which differs between normal mouse and human gene expression was *Dpcr1* (Also known as *Mucl3*), which was highly expressed in the present study’s mouse SMCs, but not expressed in human gastric SMCs [155]. The function of *Dpcr1/Mucl3* in SMCs is unknown, but is annotated to be present in the cytoplasm and serve as a transmembrane protein. The protein is likely glycosylated and could play a part in NF-κB signaling and cell growth [52]. According to the STRING database, *Dpcr1/Mucl3* interacts with *Gkn2*, which was another highly-expressed gene [48].

Other highly-expressed genes with unclear function were the long non-coding RNA (lincRNA) *Yam1*/*Gm26924* and *Gm26917* [63], as well as the small nuclear RNA (snRNA) *Rn7sk*. *Rn7sk* is known to be important for gene regulation of highly-expressed gene pairs which are co-expressed through a shared bi-directional promoter [156]. We postulate that *Gkn1/Gkn2* could be such a gene pair regulated by *Rn7sk*, since they are situated right next to each other in the genomes of both humans and mice, and they are also transcribed in opposite directions.

In the present study, mitochondrial genes were also highly expressed. *mt-Rnr1* and *mt-Rnr2* are mitoribosome rRNA [63], and the only gene with a higher TPM than these two was *Tff1*. The OXPHOS genes *mt-Atp6* and *mt-Co1* were also highly expressed.

Another category of highly-expressed genes were genes with a structural function, namely *Krt8*, *Krt19*, *Actg1*, and *Mal* [52]. Of these, the first three are intermediate filament genes. KRT8 and KRT19 likely forms a keratin pair in these SMCs, since KRT8 is a type II and KRT19 is a type I keratin, and they have similar expression levels. Co-expression of KRT8/KRT19 is previously known in other epithelial cells [157].

### The mucus produced by SMCs is glycosylated with large and heavily fucosylated glycans, and this glycosylation does not change with early Helicobacter pylori infection in mice

While the gastric mucin glycosylation is characterized by extreme heterogeneity in humans [26], there was low inter-individual variation in the present mouse data, likely due to that the mice were an inbred strain. Furthermore, while the mucin glycans differed between chronically *H. pylori*-infected and non-infected humans, and early *H. pylori* infection has been shown to increase the expression of sialylated Lewis antigens in the rhesus monkey [158], the glycans did not change as a result of early infection in the mouse. This stable glycosylation was comprised of remarkably large and heavily fucosylated glycans, and such FUT2 dependent fucosylation binds to *H. pylori* [24]. A large proportion of the terminal motifs consisted of H antigens, and in particular H type 2 antigens.

A limitation with the glycome analysis was that it was performed on corpus tissue rather than LCM-isolated SMCs. As a result, approximately 95% of detected glycans originated from MUC5AC-expressing SMCs, with a minor contribution from MUC6-expressing mucus neck cells [159]. This approach was chosen due to that in-depth glycome profiling requires ~100 µg of glycoprotein which is not feasible with LCM. O-glycomes obtained from tissues and isolated mucins are essentially identical, indicating that the glycomes reported here mainly reflects mucin glycosylation [55].

Another limitation with the present glycome study was that it was conducted on two cohorts different from the cohort which the RNA-Seq data was based on. Since 17 terminal motifs were differentially-expressed between the glycan cohorts, the glycosylation could have been different for the RNA-Seq cohort. However, the PCA and clustermap revealed that this difference was driven by a cluster of four mice in cohort G2, which had a slightly lower fucosylation. We could not find any explanation for this divergent cluster in information known to us about the mice (age, weight, experimental notes). Within this cluster of four divergent mice there were both *H. pylori*- and sham control mice. Of importance, the other four mice in that cohort had an indistinguishable glycosylation to that of cohort G1. Therefore, it is unlikely that the overall pattern, with unchanging glycosylation with *H. pylori* infection and the glycans being overall large and heavily fucosylated, would have been different in the RNA-Seq cohort. Finally, that the most abundant glycosyltransferases could neatly explain the main glycosylation pathways also lends credence to the notion that the overall glycosylation was the same between all three cohorts.

Holmén-Larsson et al. 2013 characterized gastric glycans in C57BL/6 mice using a similar method as the present study [23]. In the present study, more structures were discovered (100 vs 31), and they were also both larger and more fucosylated in comparison to Holmén-Larsson et al. 2013 [23]. In line with our observation that *Fut2* was the 4th most abundantly-expressed glycosyltransferase with activity on mucin *O*-glycans, Magalhães et al. 2009 described 40 glycan structures in the gastric mucosa glycans of C57BL/6 mice and determined that the glycans were overall large and heavily fucosylated, whereas there was almost no fucosylation in isogenic *Fut2*-null mice [24]. Furthermore, the two most abundant glycans in the present study (Fuc(α1–2)Gal(β1–3)[Fuc(α1–2)Gal(β1–4)GlcNAc(β1–6)]GalNAcol and Fuc(α1–2)Gal(β1–3/4)GlcNAc(β1–3)[Fuc(α1–2)Gal(β1–4)GlcNAc(β1–6)]Gal(β1–3)[Fuc(α1–2)Gal(β1–3/4)GlcNAc(β1–6)]GalNAcol) correspond to abundant peaks at m/z 1331 and 2404 respectively in Magalhães et. al. 2009 Figure 4 [24]. The bimodal size distribution of the glycans in the present study was likely the result of B3GNT3 competing with FUT2, where core 1 and core 2 Gal(β1–3)GalNAcol were either extended with GlcNAc(β1–3) through B3GNT3 activity, or fucosylated by FUT2, thus locking the glycans into one of the two mutually exclusive main glycosylation pathways leading to either Fuc(α1–2)Gal(β1–3)[Fuc(α1–2)Gal(β1–4)GlcNAc(β1–6)]GalNAcol or Fuc(α1–2)Gal(β1–3/4)GlcNAc(β1–3)[Fuc(α1–2)Gal(β1–4)GlcNAc(β1–6)]Gal(β1–3)[Fuc(α1–2)Gal(β1–3/4)GlcNAc(β1–6)]GalNAcol.

The *Fut2*-dependent fucosylation was also shown to be important for BabA-mediated adhesion of *H. pylori* [24], and although we found no clear evidence in the published literature that glycan size or fucosylation status affect *H. pylori*-driven carcinogenesis, the BabA adhesin is connected to gastric cancer [160]. Sialylated glycans are also important for *H. pylori* binding to mucins via the sialic acid binding adhesin (SabA) [161–163]. Additional adhesins have been identified in *H. pylori* [164], however, although some of these adhesins bind to salivary mucins, there is no observed distinct binding to human gastric mucins with these [161,163,165]. *H. pylori* strains expressing BabA and SabA bind to human mucins in a manner largely driven by mucin fucosylation, and this binding correlates with the relative abundance of α1,2-fucosylated structures on mucins from both non-infected and *H. pylori*–infected individuals [166]. It is difficult to establish how the glycan size affects binding in the complex glycan environment of mucus. On the one hand there are specific requirements/minimal epitopes determining specificity for the BabA adhesin [167], and mucin glycan size has been correlated to pathogen binding in species with relatively small mucin glycans [168]. By contrast, both human and mouse mucin glycans are large and complex and the high density of fucosylated glycans in a solution may confer both opportunities for binding that does not occur to a large extent when sparsely-located epitopes on a rigid surface are presented. Multivalency within the dense mucin-glycan environment likely also enables otherwise weak interactions to become biologically significant. In the present study with short-term infected mice, sialylated and sulfated glycans had very low abundance. In long-term infection in rhesus monkeys and humans there are increased levels of sialylated Lewis antigens as demonstrated with immunohistochemistry [158,169,170]. Meanwhile, sulfated glycans are common in gastric mucins from patients with intestinal metaplasia and gastric tumors, as shown by mass spectrometry [171,172]. There is no clear evidence in the literature on how long-term *H. pylori* infection affects the large fucosylated glycans that dominate the glycome in the present study, but increases in sialylated and sulfated structures implies a corresponding reduction in the relative abundance of other structures.

### Functional network analysis indicates that interferon stimulation might cause a reduction of oxidative phosphorylation in SMCs

The overwhelming majority of the most up-regulated genes were inducible by either type I, II, or III-interferon. These ISGs formed a functionally related cluster according to STRING database annotation and cluster analysis. This cluster was highly interconnected with another cluster of down-regulated genes related to protein synthesis, which in turn was functionally connected to a third cluster of down-regulated genes related to OXPHOS. This pattern suggests a chain of events where interferon stimulation cause a reduction of protein translation, resulting in reduced OXPHOS activity. Furthermore, the absence of any clear gene expression pattern related to cell death indicates that this OXPHOS inhibition is not driven by apoptosis or another form of cell death. Further experiments are necessary to investigate this process.

Similar findings to this proposed chain of events have been shown experimentally in another model system, a gastrointestinal mouse infection model with *Citrobacter rodentium*. Both infection with *C. rodentium* and IFN-γ stimulation caused a reduction of OXPHOS activity in the large intestine [149]. Meanwhile, the *H. pylori* infection itself leads to OXPHOS inhibition in systems independent of exogenous interferon stimulation [173]. In line with this, the *H. pylori* toxin VacA has *in vitro* been shown cause a reduction of OXPHOS activity, which was not due to cell death [174]. Essentially all *H. pylori* strains express VacA, however VacA is considered weak in the SS1 strain [175].

### The differential gene expression profile provides a snapshot of both the early H. pylori infection and early gastric precancerous cascade in mice

*H. pylori* infection causes gastritis, and when chronic, this inflammation can continue to a pre-cancerous cascade which ends in gastric adenocarcinoma [176]. Unsurprisingly, much of the differential gene expression profile from the experimental early *H. pylori* infection described in this article was related to inflammation. Apart from the up-regulated genes being dominated by ISGs as discussed above, this was also evident in the SetRank gene set enrichment results where one of the top two gene sets was related to IFN-γ stimulation, the other being related to OXPHOS. When assessing gene set enrichment analysis results, it is important to keep in mind that the only gene sets that can be highlighted by the analysis are the gene sets which the analysis was built upon [46]. One of the few curated *H. pylori* infection gene sets in existence is the KEGG pathway “hsa05120 Epithelial cell signalling in *Helicobacter pylori* infection - *Homo sapiens*,” but there is no mouse variant [42]. While this human KEGG gene-set involves the mitochondria, it is not related to OXPHOS down-regulation itself, but rather how the virulence factor VacA targets mitochondria and induces apoptosis. The gene-set further describes how VacA induces vacuolation with the help of V-ATPase, of which one of the sub-units (ATP6V1D) was differentially expressed in our data. Much of the hsa05120 gene-set pertains to the effects of CagA on inflammation, cell morphology, proliferation, motility, and apoptosis. However, the *SS1 H. pylori* strain does not have a functional type IV secretion system (T4SS) and thus cannot inject cells with CagA [177]. The dataset we have formed can form the genesis for new gene sets, thus improving future gene set enrichment analysis.

A notable absence of differentially-expressed genes in the early *H. pylori* infection were the most highly-expressed genes, which can be seen as function-defining SMC genes. Taken together with the functional network analysis results, this suggests the possibility that the SMCs performs the same functions during early *H. pylori* infection, but at a reduced rate due to OXPHOS reduction.

When examining individual genes, the inflammation-related gene *Gm23935* [139,140] was the most abundant differentially-expressed gene. Also noteworthy was *Nkx6-3*, which showed a stark depletion in *H. pylori*-infected mice and had the most extreme log2 fold change (less than −7). *Nkx6-3* is associated with the precancerous cascade, as a loss of the encoded protein, Homeobox protein Nkx-6.3, contributes to the development of gastric adenocarcinoma [146,147]. NKX6-3 suppresses *NF-kB* and *Dnmt1* [147], which in turn seems to induce *Hace1* expression and Reactive Oxygen Species (ROS) production [147]. However, in our data, there was no significant difference in gene expression for *Hace1*, *Nf-kB*, or *Dnmt1*. This may be explained by the short infection duration in the present study or suggest an alternative mechanism operating in mouse SMCs in vivo.

Even though mouse models are common for both gastric cancer and *H. pylori* infection, they do not perfectly recapitulate human pathogenesis. The major shortcoming of the various mouse gastric cancer models is the poor representation of the later stages of cancer development, but they do perform better for studying the earlier stages of the pre-cancerous cascade [178]. *H. pylori* has a host tropism toward humans and do neither readily infect mice, nor create the same level of gastritis. Mouse-adapted strains such as SS1 used in the experiment described here, are often used to better and successfully recapitulate disease phenotypes such as loss of parietal cells [178]. CagA is an important virulence factor for human *H. pylori* infection pathogenesis; however, the previously described SS1 strain does not have a functional T4SS and therefore cannot inject CagA. Furthermore, *H. pylori* strains tend to lose T4SS function when chronically infecting mice [178]. The tendency of *H. pylori* to drop CagA function when adapting to mouse hosts could indicate that CagA is not important for *H. pylori* when infecting mice. Another notable difference between the human and mouse *H. pylori* pathogenesis is the differing microbiota which can at least partially be explained by the presence of a squamous epithelia forestomach and coprophagic behavior in mice, as well as a higher pH of 3–4 rather than pH around 1 in humans [179].

### RNA-Seq of cells collected through LCM is a viable alternative to both scRNA-Seq and in situ spatial transcriptomics to study specific gastrointestinal epithelial cells in vivo

The results from the present work shared some similarity to a microarray-based LCM study of *H. pylori*-infected mice [11]. Similarly, both IFN-α and IFN-γ inducible genes and other inflammation-related genes were up-regulated during *H. pylori* infection. Relating these findings to humans, a mechanism that could contribute to IFN-α stimulation is TLR8 signaling in monocytes in response to *H. pylori* infection [180]. Another similarity in the present study to the microarray-based LCM study was the high purity of cellularity type. Dissimilarly, OXPHOS-related genes did not seem to be greatly perturbed in SMCs when subjected to *H. pylori* infection, although the specific genes included in the microarray are unavailable since the dataset where this was listed is no longer accessible, thus preventing direct comparisons. A strength of RNA-Seq as compared to microarrays is a greater coverage of the transcriptome and the ability to cover all important genes such as *Muc5Ac*, which otherwise could be absent in microarrays [11].

A limitation of LCM-derived material to analyse gene expression is the low quantity of RNA that can be purified. This can be mitigated by harvesting from more tissue sections, but this is labor-intensive and can be limited by both the total amount of tissue available and time due to the degradation of RNA. RNA amplification prior to library prep is another option, but risks introducing bias in the transcripts [11]. We opted not to amplify the RNA prior to library prep, but rather use the low-input library kit *SMARTer Total Stranded RNA-Seq, Pico input mammalian – V3* (Takara Bio, Kusatsu, Japan) to minimize bias [181]. Furthermore, this kit prepares a library independent of poly(A)tails, thereby improving the detection of differentially-expressed, non-coding RNAs.

The specificity of the LCM cellular collection method is high, but not perfect. The overwhelming majority of sequenced cells were SMCs, while a small minority were parietal and endothelial cells. There was a miniscule yet non-zero presence of marker genes for all other cell-types, and it is uncertain if this indicates a real presence of these cell types or not. Considering the histological proximity between parietal and endothelial cells to the SMCs it is plausible that they indeed were present in a small yet negligible quantity in the sequenced material.

The marker gene expression for the SMCs or parietal cells between infected and sham control samples did not differ. In contrast, among the other marker genes there was a significant difference in marker gene expression for plasma cells (Zbp1), monocytes (Tet2), and endothelial (Xdh and S100a13) cell types. Notably for the 43 endothelial cell markers, the only high-abundance gene (*Tspan8*) was not differentially expressed. However, none of the other marker genes for these cell types had a significant difference in gene expression levels between infected and sham control samples. Furthermore, these differentially-expressed marker genes are also present in other cell clusters which can be related to surface mucus cells, according to the PanglaoDB web browser. Thus, it is unclear if these marker genes indicate an actual difference in non-surface mucus cell populations that were potentially present in the sequenced tissue.

A potentially more specific collection method would be cell-sorting, and this method has been used to provide gastric mouse cells for downstream scRNA-Seq to study the pre-cancerous cascade in a chemically, rather than *H. pylori*, induced model [17,182,183]. scRNA-Seq allows the study of all viable cells that can be sorted quickly after the tissue has been removed, in contrast to just the cells that can reliably be separated with LCM which requires that the desired cell-types can be readily identified with the staining used on the slides in the microdissection apparatus. A downside with scRNA-Seq is the methodological and logistical challenge of the cell-sorting which is more difficult and time-consuming than simply snap-freezing the tissue for later processing and might thus limit experimental design for collecting the tissue. For example, the time-constraint derived from the need to avoid harvest-induced changes in gene expression limits the throughput and possibilities to combine scRNA-Seq with other experiments in the same harvest. Another downside is that scRNA-Seq remains a more expensive method than LCM and RNA-Seq combined.

Another alternative method for spatial transcriptomics is *in situ* RNA-Seq, wherein the transcriptome of a microscopy slide can currently be seen on a resolution as detailed as 0.5 µm, depending on the method [184]. This not only allows the analysis of gene expression with a cellular (or even sub-cellular) resolution, but also how the gene expression depends on where the cells are in relation to other cells and in the tissue. While this method is expensive, it might enable the exploration of research questions otherwise not possible to answer with other methodology. A substantial drawback of spatial transcriptomics is the difficulty in handling and analysing the data, which has yet to reach a state of maturity with standardized data formats, processing pipelines, and quality controls [184].

The RNA-Seq results were partially validated on the protein level with IF. Two out of three tested proteins (LGALS3BP and RPL19) differed significantly between *H. pylori*-infected and sham control mice, and there was also a significant positive correlation between protein and mRNA levels for LGALS3BP/*Lgals3bp*. Although the correlation between protein and mRNA levels for RPL19/*Rpl19* was not significant (adjusted *p* = 0.055), considering that the raw *p*-value was 0.037 and Spearman’s ρ was 0.72 the correlation between protein and gene expression would likely have been significant with a larger number of samples. In contrast there was no significant difference between the two groups in COX6C signal intensity, nor a correlation between COX6C signal and *Cox6c* gene expression. Possible explanations for the results include post-transcriptional regulation maintaining COX6C protein levels despite declining mRNA levels, that unlike the more diffuse cytoplasmic proteins LGALS3BP and RPL19, the mitochondrial protein COX6C would be unevenly distributed close to the nuclei, and there could be substantial differences in what percentage of the visible cells in each sectioned gland that consists of the mitochondrial compartment, thus introducing a degree of noise into the readout. Even though the COX6C IF did not validate the differential expression of the OXPHOS-cluster, downregulation of OXPHOS activity has been shown experimentally in *C. rodentium* infection, in vitro IFN-γ stimulation and in vitro *H. pylori* infection [149,174]. Thus, it is likely that other validation approaches could have confirmed that the downregulation of the 29 OXPHOS genes observed in this study had biological consequences. The LCM RNA-Seq workflow described in the present study contributes to a greater usefulness of *H. Pylori* mouse models, and potentially other mucosal infection models as well, by providing another feasible tool for spatial transcriptomics.

### Formation of new gene sets based upon this dataset

Gene set enrichment analysis (GSEA) and related bioinformatics analysis such as SetRank are predicated on previously established gene sets. Current gene sets related to *H. pylori* infection and/or SMCs are limited, which begs for an expansion of such gene sets. We plan to take all differentially-expressed genes presented in the current study and create a child term of “response to Gram-negative bacterium” (GO:0140460), which could be named “Mouse gastric corpus surface mucus cell response to *Helicobacter pylori* infection.”

## Conclusion

This article shows that RNA-Seq of cells collected with LCM is a viable method to investigate host-pathogen interactions *in vivo* and provides a detailed protocol for it. The hallmarks of mouse SMCs both with and without early *H. pylori* infection have been revealed in greater detail than previous studies [11,17,23–25,183]. The gene expression is dominated by genes coding for secreted proteins such as TFF1, GKN1, GKN2, and MUC5AC, mitoribosome RNA, and cytoskeleton proteins. Another gene which characterized the SMCs was *Rn7sk*, which we postulate regulates the gene pair *Gkn1/Gkn2*. Furthermore, the known murine gastric mucin glycome was expanded and shown to be dominated by large, heavily fucosylated glycans, whose biosynthesis could be accounted for by the 15 most abundant glycosyltransferases.

We found that *H. pylori* infection caused OXPHOS-related genes to be down-regulated. These genes were functionally associated with down-regulated, protein-synthesis-related genes, which then were functionally associated with up-regulated, IFN-induced genes. While the sequential steps of this hypothesis remain to be validated experimentally, the results suggest that early *H. pylori* infection elicits IFN stimulation of the SMCs *in vivo*, subsequently diminishing protein synthesis, particularly of proteins required for OXPHOS. Along this pathway there may be potential drug targets. We also found depletion of *Nkx6-3* gene expression to be one of the earliest events of the *H. pylori-*induced pre-cancerous cascade. This article contributes a piece to the greater puzzle of *H. pylori* pathogenesis, which in turn can lead to improved treatment and prevention of *H. pylori*-related death and disease in humans. Finally, the dataset can form the genesis for new gene sets, thus improving future gene set enrichment analysis.

## Data Availability

All data is freely available under permissive open licenses permitting reuse (CC-BY, CC0, or MIT). The RNA-Seq FastQ files are available at the European Nucleotide Archive (ENA) with the project accession number PRJEB70775: https://www.ebi.ac.uk/ena/browser/view/PRJEB70775 [185]. The NGI data delivery that support the findings of this study are openly available in Svensk Nationell Datatjänst (SND) at https://doi.org/10.5878/ejy9-2895, reference number 2023–166/1 [186]. The R-scripts, Jupyter notebook, and other files used for the data analysis is available on GitHub: https://github.com/mattias-erhardsson/lmpc-infection-rnaseq [187]. The GitHub repository has been archived at Zenodo: https://doi.org/10.5281/zenodo.18151929 [188]. Supplemental files, including the ARRIVE checklist, are available in the same GitHub repository as above: https://github.com/mattias-erhardsson/lmpc-infection-rnaseq/tree/main/Virulence%20supplemental%20files [185]. The glycomics data are available in GlycoPost (https://glycopost.glycosmos.org/) at http://doi.org/10.50821/GLYCOPOST-GPST000464 [189].

## Open scholarship

This article has earned the Center for Open Science badges for Open Data and Open Materials through Open Practices Disclosure. The data and materials are openly accessible at https://doi.org/10.5878/ejy9-2895
